# Adaptive survival strategies of rumen microbiota with solid diet deficiency in early life cause epithelial mitochondrial dysfunction

**DOI:** 10.1093/ismejo/wraf064

**Published:** 2025-04-06

**Authors:** Shiqiang Yu, Yuting Fu, Jinrui Qu, Kai Zhang, Weiyun Zhu, Shengyong Mao, Junhua Liu

**Affiliations:** Laboratory of Gastrointestinal Microbiology, Jiangsu Key Laboratory of Gastrointestinal Nutrition and Animal Health, College of Animal Science and Technology, Nanjing Agricultural University, Nanjing 210095, China; National Center for International Research on Animal Gut Nutrition, Nanjing Agricultural University, Nanjing 210095, China; Laboratory of Gastrointestinal Microbiology, Jiangsu Key Laboratory of Gastrointestinal Nutrition and Animal Health, College of Animal Science and Technology, Nanjing Agricultural University, Nanjing 210095, China; National Center for International Research on Animal Gut Nutrition, Nanjing Agricultural University, Nanjing 210095, China; Laboratory of Gastrointestinal Microbiology, Jiangsu Key Laboratory of Gastrointestinal Nutrition and Animal Health, College of Animal Science and Technology, Nanjing Agricultural University, Nanjing 210095, China; National Center for International Research on Animal Gut Nutrition, Nanjing Agricultural University, Nanjing 210095, China; Laboratory of Gastrointestinal Microbiology, Jiangsu Key Laboratory of Gastrointestinal Nutrition and Animal Health, College of Animal Science and Technology, Nanjing Agricultural University, Nanjing 210095, China; National Center for International Research on Animal Gut Nutrition, Nanjing Agricultural University, Nanjing 210095, China; Laboratory of Gastrointestinal Microbiology, Jiangsu Key Laboratory of Gastrointestinal Nutrition and Animal Health, College of Animal Science and Technology, Nanjing Agricultural University, Nanjing 210095, China; National Center for International Research on Animal Gut Nutrition, Nanjing Agricultural University, Nanjing 210095, China; Laboratory of Gastrointestinal Microbiology, Jiangsu Key Laboratory of Gastrointestinal Nutrition and Animal Health, College of Animal Science and Technology, Nanjing Agricultural University, Nanjing 210095, China; National Center for International Research on Animal Gut Nutrition, Nanjing Agricultural University, Nanjing 210095, China; Laboratory of Gastrointestinal Microbiology, Jiangsu Key Laboratory of Gastrointestinal Nutrition and Animal Health, College of Animal Science and Technology, Nanjing Agricultural University, Nanjing 210095, China; National Center for International Research on Animal Gut Nutrition, Nanjing Agricultural University, Nanjing 210095, China

**Keywords:** solid diet deficiency, rumen microbiota, survival strategies, ruminal epithelium, mitochondrion, energy

## Abstract

With extreme nutritional substrate deficiency, the adaptive responses of the gastrointestinal microbiota and host metabolism are largely unknown. Here, we successfully established a microbial substrate deficiency model in the rumen without solid diet introduction in neonatal lambs. In the absence of solid diet, we observed a reduction in the Simpson Index of rumen bacteria, along with a marked decline in the abundance of keystone microorganisms such as *Prevotella*, *Selenomonas*, *Megasphaera*, and *Succiniclasticum*, indicating a simplified microbial interaction network. Additionally, more urea and NH_3_-N production facilitated microbial efficient nitrogen utilization to prioritize ammonia as a nitrogen source for survival, reallocating energy to overcome nutritional limitations and sustain their viability. In addition, enriched archaea (*Methanosarcina*, *Methanomicrobium*, *Methanobrevibacter*, and *Methanobacterium*) promoted hydrogen removal and the growth of nitrogen-producing microorganisms (*Pecoramyces*, *Piromyces*, *Caecomyces*, and *Orpinomyces*). It also reinforced the glutamate-glutamine pathway, as evidenced by the higher expression of *glnA*, *GLUL*, *gdhA*, and *ureAB*, suggesting enhanced internal cycling of nitrogen for microbial survival. This selfish microbial survival strategy deprived the host of adequate volatile fatty acids for energy metabolism, resulting in the downregulation of rumen epithelial cell cycle proteins (*CCNB1*, *CCNE*), abnormal mitochondrial morphology, and reduced mitochondrial deoxyribonucleic acid copy number and adenosine triphosphate production. Overall, these findings revealed the adaptive survival strategies of rumen microbiota with solid diet deficiency in early life, which caused alterations in epithelial cell mitochondrial function.

## Introduction

Microorganisms are found throughout diverse ecosystems across the earth and represent the oldest, most phylogenetically diverse, and most widely distributed forms of life on the planet [1]. However, competition for limited resources is intense among microorganisms, often leading to situations where energy supply barely meets their basic metabolic needs, pushing many to the brink of survival [2–4]. In extreme environments, such as high-temperature volcanic vents and arid deserts, microorganisms often experience nutritional “starvation”, where cellular maintenance and proliferation are severely restricted [5, 6]. Under such conditions, microorganisms must adjust their metabolic pathways and processes to survive. Similarly, even within the resource-rich environment of the animal gut, microorganisms must adapt to conditions of extreme nutrient excess or scarcity to successfully colonize and thrive within the host’s gastrointestinal tract [7–9]. Although the impact of these microbial adaptations is increasingly recognized, the adaptive responses of the gut microbiota under oligonutrient conditions, along with the associated metabolic alterations in the host, remain intriguing mysteries that require further investigation.

Confronted with nutrient and energy limited environments, microorganisms have evolved an extensive array of adaptations. Their energy metabolism is up to 100 times lower than that of normal microorganisms, and oligonutrient organisms sacrifice growth rates to optimize resource utilization [10, 11]. These adaptations can extend lifespan and maximize survival in energy-limited environments. Under such conditions, dispersal is limited, and the rate of resource supply is regulated by endogenous processes, including the internal cycling of nutrients and energy [6]. Moreover, it has been observed in laboratory culture media that microorganisms may prioritize ammonia as a nitrogen source for survival, reallocating energy to overcome nutritional limitations and sustain their viability [12, 13]. Microorganisms also coordinate behaviors through quorum sensing, such as biofilm formation, exoenzyme production to degrade complex organic matter, and metabolic pathway regulation, to more effectively utilize the limited resources available in the environment [14, 15]. During adaptation to extreme nutritional conditions, niche-specific adaptations enable microorganisms to maintain their status as operational units, providing a competitive advantage over other species [14, 16]. Current animal research primarily focuses on the consequences of reshaping the gut microbiome due to deficiencies in trace elements or specific nutrients [8, 17]. However, there is limited exploration of the life strategies of microorganisms and the host’s adaptive responses under conditions of extreme starvation. This gap hinders our understanding of microbiome dynamics in gut ecosystems under nutrient-deficiency conditions.

Over a long evolutionary process, ruminants have evolved a complex digestive system known as the rumen fermentation vat [18]. The presence of the rumen circumvents the dietary-microbe interaction limitations faced by monogastric animals due to the powerful enzymatic digestion in their foregut [19]. In addition, the digestive system of neonatal ruminants gradually transitions from a monogastric digestion mode to a rumen-based system, a unique adaptation not observed in other mammals [20, 21]. During this transition, liquid food can bypass the rumen and inflow into the abomasum via the esophageal groove, whereas solid food enters the rumen directly [22, 23]. By leveraging this characteristic, we can explore the adaptive responses of the rumen microbiota under extreme nutrient substrate deficiency conditions. In this study, we established a rumen microbial substrate deficiency model by withholding solid diet introduction in neonatal lambs. We observed that during early life deprivation of a solid diet, the rumen microbiota exhibited selfish behavior, limiting the production of additional energy substrates for the host. This adaptive strategy led to compromised mitochondrial energy metabolism in the rumen epithelial cells. These findings revealed a self-serving nutritional competition strategy among rumen microorganisms and provided direct evidence of microbial self-preservation and host adaptive changes under nutrient-deficiency conditions.

## Materials and methods

### Animal experiment and sampling

The animal experiment was conducted according to the guidelines of the Animal Care and Use Committee at Nanjing Agricultural University (SYXK2017–0027).

Sixteen 11-day-old healthy sucking twin lambs with similar initial body weights (5.88 ± 0.76 kg) were selected. After three-day adaptation, they were then subjected to different feeding strategies: the nutritionally enriched group received milk + corn-soybean starter + alfalfa hay (CON, *n* = 8), whereas the solid diet deficiency group received only milk (LN, *n* = 8). Milk feeding was conducted using bottles to ensure the passage of milk through the esophageal groove into the abomasum. The lambs of CON group were provided with 600 ml/day of goat’s milk, which was prepared from goat milk powder (water: goat milk powder = 10:1) and equivalent to 10% of their initial average body weight [24]. And lambs in the LN group were provided with unrestricted access to goat milk. The mixed goat milk formula was administered four times daily at specific intervals (07:00, 12:00, 17:00, and 22:00), while solid diet was given twice daily, at 08:00 and 17:00. The solid diets used in the experiment were analyzed for dry matter (method 930.15), crude protein (method 973.48), ether extract (method 920.39), and crude ash (method 942.05) following AOAC [25] protocols. Neutral detergent fiber and acid detergent fiber were sequentially determined using an Ankom200 fiber analyzer (ANKOM Technology Corp., Macedon, NY) with the ANKOM filter bag method. The nutritional composition of both solid diets is provided in Supplementary Table S1.

At 42 days of age, after 3–4 h morning feeding, the lambs were slaughtered, and the rumen contents were meticulously collected and homogenized. The rumen fluid sample was filtered through four layers of gauze and stored at −80°C for subsequent metabolite analysis. Additionally, 10 g rumen content sample was stored at −80°C for microbial deoxyribonucleic acid (DNA) extraction. The rumen epithelium was rinsed, flash-frozen in liquid nitrogen, and stored for subsequent RNA extraction.

### Measurement of ruminal volatile fatty acids, ammonia nitrogen, and microbial crude protein levels

As previously described, the concentrations of volatile fatty acids (VFAs) were analyzed using gas chromatography (GC-7890B; Agilent Technologies, Santa Clara, CA) [26]. The ruminal ammonia nitrogen (NH_3_-N) concentration was determined using a microplate reader (Multiskan FC, Thermo Fisher Scientific, New York, USA) following the method as previously described [27]. The ruminal microbial crude protein (MCP) content was quantified using the colorimetric method [28].

### Microbial deoxyribonucleic acid extraction and 16S ribosomal ribonucleic acid sequencing of bacteria attached to the diet

Microbial DNA was extracted using the E.Z.N.A. Soil DNA Kit (Omega Bio-Tek, USA) following the manufacturer’s instructions. The quantity and quality of the extracted DNA were assessed using the ND-1000 spectrophotometer (NanoDrop, Wilmington, DE, USA) and confirmed by electrophoresis on a 1% agarose gel. The DNA samples were subsequently stored at −80°C for further sequencing analysis. The V3-V4 region of the 16S ribosomal ribonucleic acid (rRNA) gene was amplified using universal primers (341F [5′-CCTAYGGGRBGCASCAG-3′] and 806R [5′-GGACTACNNGGGTATCTAAT-3′]). Following amplification, the products were purified using the QIAquick polymerase chain reaction (PCR) Purification Kit (Qiagen, Hilden, Germany), and libraries were constructed following Illumina’s guidelines (Illumina, San Diego, CA). All libraries were sequenced using the MiSeq PE-250 platform (Illumina, San Diego, CA). After paired-end sequencing of the validated amplicon libraries on the NovaSeq PE-250 platform (Illumina, San Diego, CA), the reads were merged and assembled using FLASH (http://ccb.jhu.edu/software/FLASH/). Quality filtering was performed using FastQC (https://www.bioinformatics.babraham.ac.uk/projects/fastqc/), and chimera sequences were identified and removed using UCHIME (http://www.drive5.com/usearch/manual/uchime_algo.html). Taxonomic classification was performed using available plugins in QIIME2. Specifically, the DADA2 plugin was employed to assess sequence quality and denoise the read data, generating an abundance table of amplicon sequence variants (ASVs). To annotate the taxonomic information of the ASVs, the classify-sklearn plugin was used to train a feature classifier based on the SILVA database (version 138.2), with the naive Bayes classifier employed for final classification.

### Metabolomics detection and analysis

Analysis of metabolites in rumen fluid using UPLC-Q-TOF/MS method. The specific detection conditions and methods were consistent with those employed in the previous study [29]. Specifically, 20 μl of rumen fluid sample was vortex-mixed with 200 μl of methanol for 30 s, followed by incubation at −20°C for 1 h. Subsequently, the samples were centrifuged at 18 000 rpm and 4°C for 15 min. The supernatant (100 μl) was collected and evaporated to dryness at room temperature. The sample was then dissolved in 100 μl of methanol (LC–MS grade, Merck). LC separation was performed on Atlantis T3 (100 × 2.1 mm, 3.0 μm; Waters) using a gradient of solvent A (5 mM ammonium formate and 0.05% formic acid buffer) and solvent B (acetonitrile) for compounds with lower polarity. Subsequently, 5 μl of sample was injected at a flow rate of 0.25 ml/min. The gradient was set as follows: 0–3 min, 5% B; 3–8 min, 5%–65% B; 8–10 min, 65%–95% B; 10–12.5 min, 95% B; 12.5–13 min, 95%–5% B; 13–17 min, 5% B. For polar compounds, LC separation was performed on an XBridge BEH Amide column (4.6 × 100 mm, 3.5 μm; Waters) using a gradient of solvent A (15 mM ammonium acetate and 0.3% ammonia buffer, pH = 9) and solvent B (acetonitrile). After injection of 5 μl sample, the flow rate was set at 0.4 ml/min. The gradient was programmed as follows: 0–1 min, 85% B; 1–12 min, 85%–30% B; 12–13 min, 30% B; 13–14 min, 30%–85% B; 14–27 min, 85% B. The mass spectrometer (Agilent 6545XT Q-TOF, Palo Alto, CA, USA) was operated with a spray voltage of −3.5 kV in negative mode and + 4 kV in positive mode. The drying gas flow rate was set to 9 L/min, and the sheath gas flow rate was set to 110 L/min. The temperature of the sheath gas was maintained at 325°C. Fast data-dependent acquisition (DDA) MS/MS experiments were conducted using a collision energy map covering the mass range of 50–1000 m/z. Peak picking and alignment were performed using Progenesis QI (Nonlinear Dynamics, Newcastle, UK). Metabolite identification was accomplished by matching acquired precursor and fragment ions with the Human Metabolome Database (http://www.hmdb.ca/), MassBank (http://www.massbank.jp/index.html), and METLIN (http://metlin.scripps.edu/index.php) databases. Data normalization and analysis were conducted using the web-based tool MetaboAnalyst 6.0 (https://www.metaboanalyst.ca/), which was also employed for metabolite enrichment analysis. Peak area data were processed by median normalization, log transformation, and auto-scaling. Differential metabolites were determined using log2(FC)>1.5, VIP>1 and false discovery rate (FDR) adjust *P*-value < .05 via Wilcoxon rank-sum test, along with Benjamini and Hochberg multiple testing correction.

### Ruminal microbial deoxyribonucleic acid extraction, metagenomic sequencing and bioinformatic analysis

Genomic DNA from microorganisms in rumen contents was extracted using DNA extraction kits (E.Z.N.A. soil DNA kit; Omega Bio-Tek, USA) following the manufacturer’s protocols. The quantity and quality of DNA samples were evaluated using an ND-1000 spectrophotometer (NanoDrop, Wilmington, DE, USA) and confirmed via examination on a 1% agarose gel. To achieve an average fragment size of 400 bp, genomic DNA was fragmented using a Covaris M220 (Gene Company Limited, China). Paired-end library construction was carried out using the NEXTFLEX Rapid DNA-Seq Kit (Bioo Scientific, Austin, TX, USA). Following library pooling, sequencing was conducted on a HiSeq X Ten platform (Illumina, San Diego, CA) using 2 × 150 bp sequencing. And sequences with over 50% of bases having quality scores <20 or containing more than 10% unidentified nucleotides were excluded. Additionally, Bowtie2 [30] was utilized to eliminate reads containing adaptor contaminants and sequences originating from the sheep host. Following this, the sequence data underwent assembly via MEGAHIT [31]. For assembly from individual samples, k-mers ranging from 21 to 99 were generated. The unmapped reads from each sample were merged for a comprehensive re-assembly using MEGAHIT, resulting in a composite assembly. Following the assemblies, comprehensive de novo assembly statistics were assessed by re-aligning singleton reads using BWA [32]. MetaGeneMark [33] was utilized to predict contigs (>500 bp) from each sample, extracting the open reading frames (ORFs) from the assembled contigs. Subsequently, CD-HIT [34] was employed for clustering, generating a non-redundant dataset with a sequence identity cut-off set at 95% and a read coverage threshold of 90%. The obtained reads were aligned to the non-redundant gene dataset using SOAPaligner [35] to calculate gene abundance, employing a similarity threshold of 95%. Additionally, all non-redundant sequences were aligned against the GenBank non-redundant database utilizing Diamond (version 0.8.35; https://www.diamondsearch.org/index.php), setting an E value cut-off at E < 1 × 10^−5^ [36]. To acquire potential functional insights and details on carbohydrate-active enzymes (CAZy), annotations were obtained from the Kyoto Encyclopedia of Genes and Genomes (KEGG) database (version 0.8.35; https://www.diamondsearch.org/index.php) and the CAZy database (https://www.cazy.org/). Principal coordinate analysis (PCoA) was conducted using Bray–Curtis dissimilarity matrices. Subsequently, all *P*-values underwent correction for FDR utilizing Benjamini and Hochberg’ s method.

### Extraction of rumen epithelial ribonucleic acid and processing for transcriptome sequencing

We employed the Trizol reagent kit (Invitrogen, Carlsbad, USA) to isolate total RNA from the rumen epithelial samples, adhering strictly to the manufacturer’s guidelines. Concentration and purity were determined using NanoDrop (NanoDrop Technologies, Wilmington, DE, USA). Integrity was assessed using the RNA Nano 6000 Assay Kit (Agilent Technologies, CA, USA). Subsequently, 1000 ng of RNA was selected for cDNA library construction. Sequencing libraries were generated using the NEBNext Ultra RNA Library Prep Kit (NEB, USA) according to the manufacturer’s instructions. Library fragments were purified using the AMPure XP system (Beckman Coulter, Beverly, USA), selecting cDNA fragments of 240 bp in length, followed by PCR amplification and purification using the AMPure XP system. Finally, library quality was assessed using the Agilent 2100 Bioanalyzer (Agilent Technologies, Palo Alto, CA, USA). Library preparation was sequenced on the MiSeq System (Illumina, San Diego, CA), generating paired-end reads.

Clean reads were obtained by removing low-quality reads (reads with adaptor sequences and reads including 0.5% unknown bases in raw reads). Mapping to the Ovisaries reference genome (Oar v3.1) was performed using TopHat (http://tophat.cbcb.umd.edu/; v2.0.9). Gene expression levels were calculated as fragments per kilobase of transcript per million mapped reads (FPKM). Differential gene expression (DEGs) was determined by comparing the two groups using the DESeq R package (1.10.1). DEGs were identified with Benjamini and Hochberg multiple testing correction [fold-change (FC) of >1.5 or < −1.], with FDR < 0.05. GO enrichment analysis of DEGs was performed using DAVID (version 6.8).

### Determination of ruminal epithelial gene expression and mitochondrial function

The total RNA extract, cDNA synthesis, and real-time quantitative PCR were performed according to previous study [37]. Subsequently, qRT-PCR was performed for all genes using the QuantStudio 7 Flex Real-Time PCR system (Applied Biosystems, Foster City, CA, USA) with SYBR green fluorescent dye detection. The amplification conditions were as follows: initial denaturation at 95°C for 30 s, followed by 40 cycles of denaturation at 95°C for 5 s, annealing at 60°C for 34 s, extension at 72°C for 60 s, and final extension at 72°C for 15 s. In this study, we concentrated on the genes related to the epithelial cell cycle. The specific genes and their sequences are detailed in Supplementary Table S3. The primers were designed using Primer 5.0 software (Whitehead Institute, USA).

Additionally, the mitochondria respiratory chain complex I, II, III, and IV assay kit (Nanjing Jiancheng Bioengineering Institute, Nanjing, China) was used to measure the concentration of mitochondrial respiratory chain enzymes I, II, III, and IV in the epithelium. The Adenosine Triphosphatase Assay Kit (Nanjing Jiancheng Bioengineering Institute, Nanjing, China) was used to detect the concentration of adenosine triphosphate (ATP) in the mitochondria. The Mitochondrial DNA Extraction Kit (PHYGENE, Fuzhou, China) was used to measure the content of mtDNA. The Tissue Reactive Oxygen Species (ROS) Test Kit (Beijing Baiaolaibo Technology Co., Ltd, Beijing, China) was used to measure ROS levels. The NAD^+^/NADH Assay Kit with WST-8 (Beyotime, Shanghai, China) was used to measure the levels and ratio of NAD^+^ and NADH. Observation of epithelial cell morphology was conducted following the method as previously described [38]. Transmission electron microscopy (Hitachi H-7650, Hitachi Technologies, Tokyo, Japan) was used to examine ultrathin sections. Specifically, epithelial samples were fixed in glutaraldehyde to maintain their morphology and structure, followed by dehydration, impregnation, gradual acetone infiltration, and embedding in resin. The samples were then sliced into very thin sections (70–100 nm) using an ultramicrotome (Leica EM FC7 Cryo Ultra-microtome, Leica Microsystems, Wetzlar, Germany). The samples were stained with uranyl acetate and lead stain to enhance the contrast of cellular structures. The stained ultrathin sections were placed on TEM grids, and the parameters such as accelerating voltage and objective magnification were adjusted on the transmission electron microscope to obtain optimal image quality.

### Statistical analysis

Unless stated otherwise, all experimental data were analyzed using SPSS software (version 25; IBM Corp., Armonk, NY, USA). Diet was considered a fixed effect, while lambs were treated as a random effect, with the significance threshold set at *P* ≤ 0.05. The normality of the untransformed data distribution was evaluated using the Shapiro–Wilk test. For data assumed to follow a normal distribution, independent sample t-tests were conducted; for variables not meeting this assumption, the Kruskal–Wallis test was applied. Pathway enrichment analysis of differential metabolites was performed using MetaboAnalyst 6.0 (https://www.metaboanalyst.ca/). Visualization and KEGG enrichment analyses of DEGs in the transcriptome were conducted using appropriate R packages [39]. Spearman correlation test was employed to assess the correlation between variables, and visualization was carried out using the corrplot and pheatmap R packages.

## Results

### Rumen microbes adapt to nutrient substrate scarcity by reshaping microbial community structure and composition

**Figure 1:**
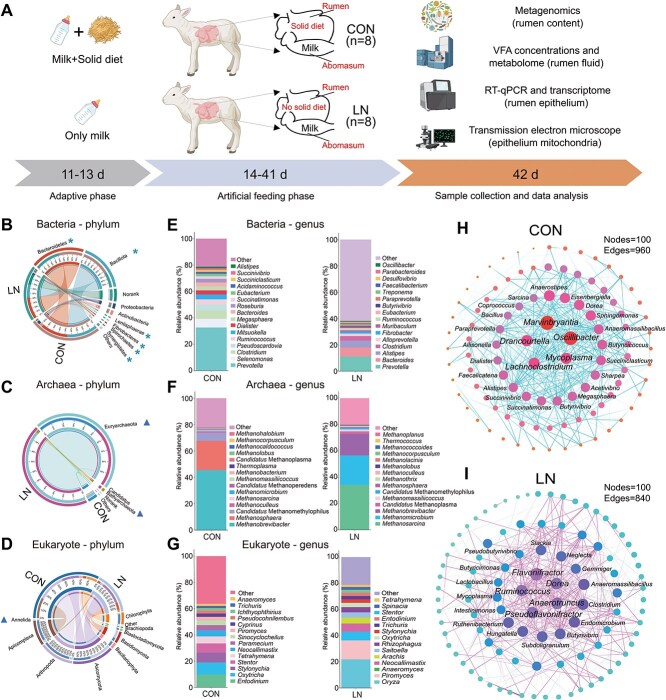
Composition and structure of rumen microbial community. A: Experimental study design, Hu lambs were randomly assigned to two groups following diets: CON, nutritionally enriched group (*n* = 8); LN, solid diet deficiency group received only milk (*n* = 8). Rumen microbiota composition at the phylum level (B: Bacteria, C: Archaea, D: Eukaryotes). Rumen microbiota composition at the genus level (E: Bacteria, F: Archaea, G: Eukaryotes). Core microbial network interactions at the genus level (H: CON group, I: LN group). Asterisks denote higher microbial abundance in CON group, while triangle denote higher microbial abundance in LN group (*P* < .05).

We established a microbial nutritional substrate deficiency model without solid diet introduction in the rumen of neonatal lambs (Fig. 1A). In this model, lambs consuming a solid diet were provided 600 ml of milk daily, while those without solid diet introduction had 1830.53 ± 92.41 ml of milk intake per day. Lambs with access to solid diet exhibited significantly higher weight gain compared to those without solid diet introduction (Supplementary Table S2). To investigate the changes in the rumen microbiome structure and function under conditions of solid diet deprivation, we employed metagenomic sequencing of the ruminal contents. A total of 5699.0 million pairs of raw reads were generated, which were subsequently processed to yield 4362.5 million pairs of high-quality reads. We observed significant separation of rumen microbiota at the domain level in PCoA plots based on Bray-Curtis distance, with bacterial abundance and Simpson Index decreasing, while archaeal abundance surged (Supplement Fig. S1A–C). Similar patterns of significant separation were observed at both the phylum and genus levels of bacteria, archaeal and eukaryote in the PCoA plots (Supplement Fig. S2A–F). Specifically, the abundance of *Bacteroidetes*, *Bacillota*, *Lentisphaerae*, *Fibrobacteres*, *Spirochaetes*, and *Synergistetes* drastically declined in the rumen lacking solid diet, whereas *Euryarchaeota*, *Candidatus* bathyarchaeota, and *Annelida* significantly increased (Fig. 1B–D). At the genus level, the dominant microorganisms under normal nutritional conditions were *Prevotella*, *Methanobrevibacter*, and *Entodinium* for bacteria, archaea, and eukaryotes, respectively. In contrast, in the absence of solid diet, the dominant genera shifted to *Prevotella*, *Methanosarcina*, and *Oryza*. Additionally, the percentage of keystone bacteria in the rumen, such as *Prevotella*, *Selenomonas*, *Megasphaera*, and *Succiniclasticum*, was significantly diminished under conditions lacking solid diet (Fig. 1E–G). This indicates a substantial shift in microbial interactions within the rumen under conditions of nutrient deficiency. Moreover, in the normal model, the core rumen microbial members are primarily represented by *Marvinbryantia*, *Drancourtella*, *Oscillibacter*, *Mycoplasma*, and *Lachnoclostridium* (Fig. 1H). In contrast, the core microbial taxa under nutrient-deficiency conditions mainly consist of *Flavonifractor*, *Dorea*, *Ruminococcus*, *Anaerotruncus*, and *Pseudoflavonifractor* (Fig. 1I). Additionally, the degree of microbial interaction within the rumen attenuated under nutrient-deficiency conditions (Supplementary Table S5). In addition, we analyzed the microbial composition of the solid diets and found that the microorganisms attached to the diets were almost entirely different from those in the rumen of lambs received solid diets (Supplementary Fig. S2G–I). This indicates that the differences observed in the ruminal microbial community is not primarily shaped by the microorganisms originating from the diets.

**Figure 2:**
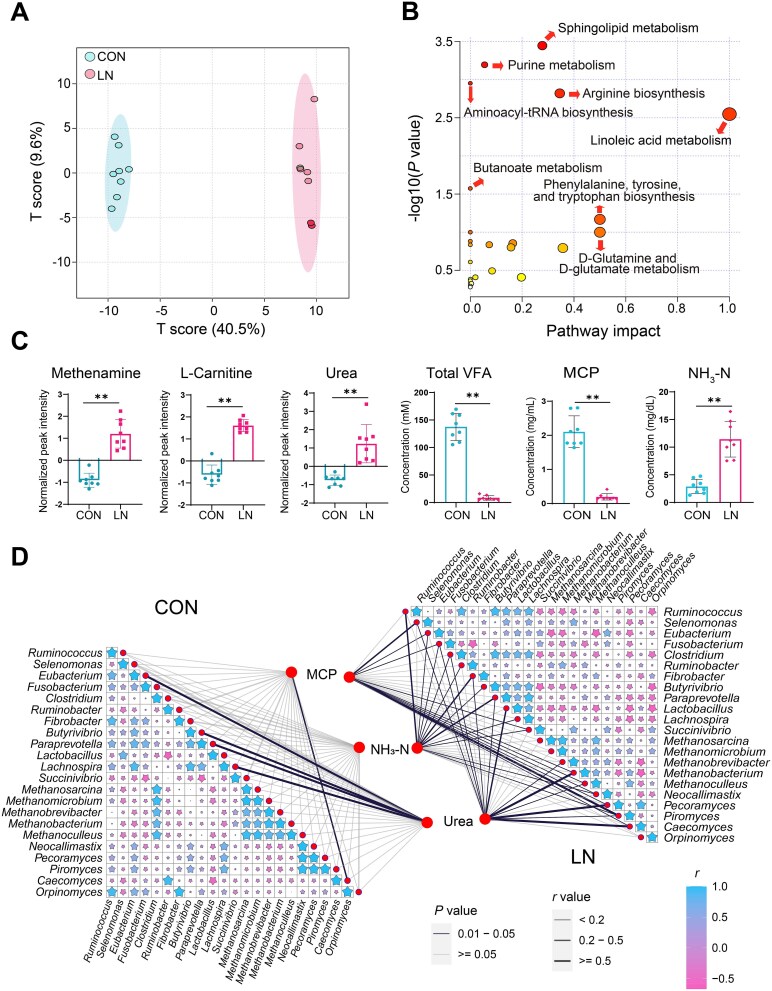
Microbial adaptive changes altered the metabolic flux in the rumen. A: Scores plot of OPLS-DA for rumen metabolites; B: Metabolic pathway analysis based on differential abundant rumen metabolites. C: Comparison of fermentation parameters and metabolites in the rumen. The concentrations of methenamine, L-carnitine, and urea were assessed using the Wilcoxon rank-sum test, with Benjamini–Hochberg correction applied for multiple comparisons. A fold-change threshold of 2 and an FDR < 0.05 were established as significance thresholds. D: Comparison of correlations and interactions among MCP, NH_3_-N, urea and energy metabolism microbiota. “*” represents *P* < .05, “**” represents *P* < .01; CON, nutritionally enriched group; LN, solid diet deficiency group received only milk. The data are presented as the mean ± SEM.

We observed at the genus level that bacteria primarily involved in protein degradation (*Selenomonas*, *Fusobacterium*, *Lactobacillus*, *Lachnospira*, and *Succinivibrio*; Supplement Fig. S3A) were reduced, while archaea utilizing hydrogen metabolism for energy production (*Methanosarcina*, *Methanomicrobium*, *Methanobrevibacter*, *Methanobacterium*; Supplement Fig. S3B) and eukaryotes metabolizing urea to produce ammonia (*Pecoramyces*, *Piromyces*, *Caecomyces*, *Orpinomyces*; Supplement Fig. S3C) were significantly enriched in the nutrient-deficiency rumen. Moreover, we observed the presence of unique archaea and eukaryotes in the rumen, such as *Candidatus* korarchaeum and *Sulfolobus*, which are typically found in extreme environments and associated with energy metabolism (Supplemental Fig. S4).

### Microbial adaptive changes alter the metabolic flux in the rumen

Orthogonal partial least squares-discriminant analysis (OPLS-DA) of annotated metabolites showed a qualitative difference between the two groups (Fig. 2A). Additionally, 77 differential metabolites were identified between the two groups [Log2(FC)>1.5, VIP>1, *P* < 0.05, Supplementary Table S4]. Furthermore, these differential metabolites were mainly associated with amino acid metabolism, linoleic acid metabolism, and butanoate metabolism according to KEGG pathway enrichment analysis (Fig. 2B). Upon specific observation, we found that the concentrations of L-carnitine and methenamine, which are closely related to bacterial growth metabolism, significantly surged under solid diet deficiency conditions, as did urea, which can serve as a nitrogen source for microorganisms, leading to a significant increase in NH_3_-N production in the rumen (Fig. 2C). Conversely, the concentrations of total VFAs (including acetate, propionate, butyrate, isobutyrate, valerate, and isovalerate) and MCP in the rumen were minimal, reflecting the scarcity of microbial-produced metabolites available for host utilization under nutrient-deficiency conditions (Fig. 2C and supplementary Fig. S5). When exploring the correlation between bacteria that decompose proteins, archaea that utilize hydrogen metabolism for energy production, eukaryotes that metabolize urea to produce ammonia, and major nitrogenous compounds (urea, NH_3_-N, MCP), strong correlations with nitrogen were found under solid diet deficiency conditions, whereas similar observations were not made under normal nutritional conditions (Fig. 2D). This suggests that the metabolic flux in the rumen is directed towards the production and utilization of ammonia rather than the production of more VFAs.

### Ruminal microbial interactions promote survival through enhanced energy and ammonia generation under solid diet deficiency conditions

**Figure 3:**
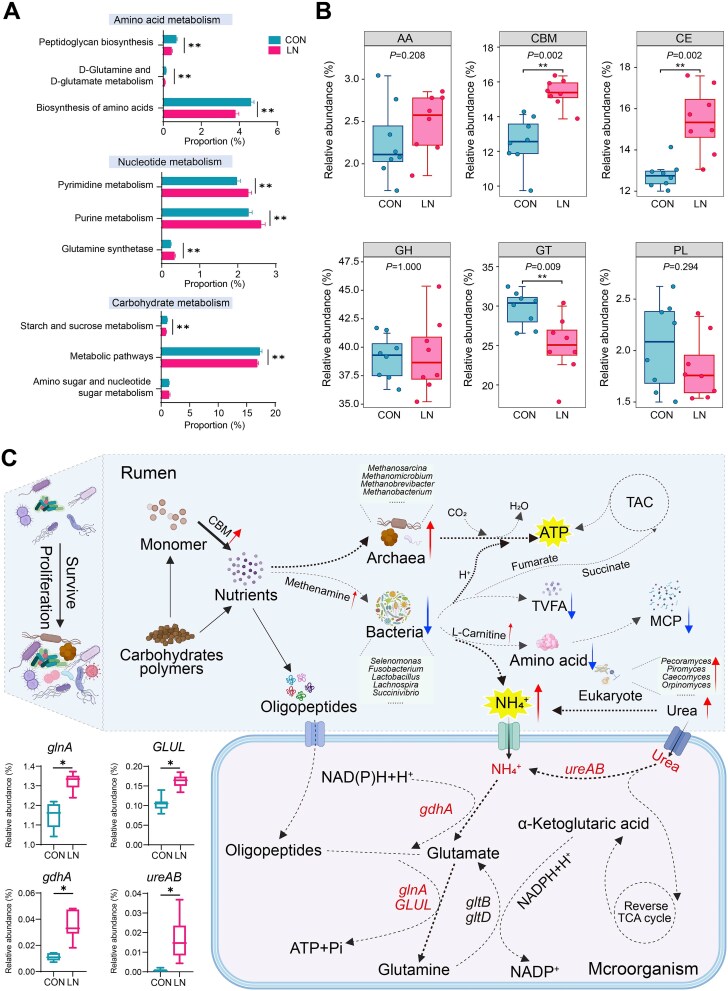
Microbial functional changes and adaptation processes under solid diet deficiency conditions in the rumen. A: KEGG functional pathways at the level 3. B: Abundance of carbohydrate enzymes at the class level. C: The survival strategy of microorganisms under solid diet deficiency conditions. “*” represents *P* < .05, “**” represents *P* < .01. CON, nutritionally enriched group; LN, solid diet deficiency group received only milk. The data are presented as the mean ± SEM.

The lack of solid diet may cause an adaptive evolution in the energy metabolism strategies of rumen microorganisms. Specifically, we found significant differences in microbial pathways at KEGG level 1, with more pathways related to microbial symbiosis and homeostasis being expressed under solid diet deficiency conditions (Supplementary Fig. S6). In more detail, pathways related to amino acid metabolism (peptidoglycan biosynthesis, D-glutamine and D-glutamate metabolism, and biosynthesis of amino acids) and carbohydrate metabolism (starch and sucrose metabolism and metabolic pathways) exhibited the lowest frequency of expression. Conversely, the expression of pathways related to pyrimidine metabolism, purine metabolism, and glutamine synthetase was enhanced (Fig. 3A). This may be described as a redistribution of resources in extreme nutrient-deficient environments, allowing microorganisms to use precursors of DNA synthesis (nitrogen) as a nutritional source to enhance their self-sustaining survival and adaptation capabilities [40]. Additionally, we also focused on the abundance changes of microbial enzymes and found that the rumen lacking nutrient substrates exhibited abundant carbohydrate-binding modules (CBMs) and carbohydrate-active enzymes (CE), which enhanced the binding rate and metabolic efficiency of rumen enzymes with substrates (Fig. 3B). Furthermore, we found significant differences in 6 auxiliary activities (AAs), 42 CBMs, 12 CEs, 62 glycoside hydrolases (GHs), 22 GTs, and 9 polysaccharide lyases (PLs) families at the level of carbohydrate enzyme families (Supplementary Table S6). The dynamic changes in these microbial enzymes indicate that the lack of solid diet alters the rumen’s micro-ecological environment, suggesting a potential reorganization in the pattern of nutrient degradation and utilization within the rumen.

We summarized the adaptation survival strategy of rumen microorganisms under substrate deficiency conditions (Fig. 3C). In the absence of solid diet, rumen microorganisms alter their energy production pathways, producing more CMB enzymes to aid nutrient acquisition. Enriched archaea (Supplementary Fig. S7) accelerate hydrogen metabolism in the rumen, generating more ATP for cellular use. Additionally, increased levels of methanamine and L-carnitine may inhibit bacterial growth, reducing the production of VFAs and MCP. Together with enriched eukaryotes, they produce more ammonia, which may serve as a preferred nitrogen source for microbial growth and energy synthesis. Additionally, we also focused on the key role of the glutamine-glutamate cycle in nitrogen metabolism and carbon metabolism, with the upregulation of *gknA*, *adhA*, *GLUL*, and *ureAB*, which promoted the synthesis of glutamine and glutamate, generating more ATP to ensure microbial energy supply. In summary, in environments of nutrient scarcity, the rumen microbial community can evolve new ecological niches, obtaining essential nutrients through cross-feeding interactions with prototrophic organisms within its microbial community, thus surviving.

### Solid diet deficiency leads to dysregulation of ruminal epithelial mitochondrial function

**Figure 4:**
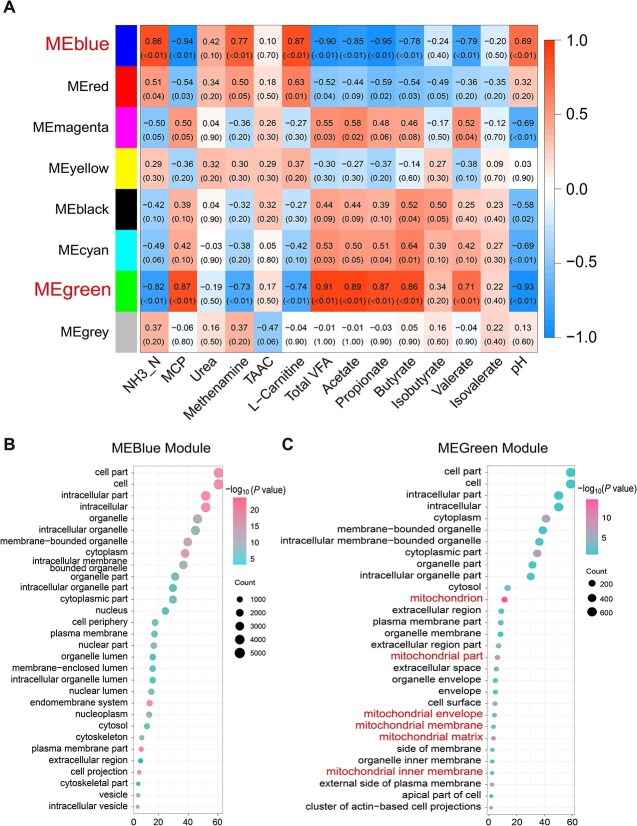
Identification of rumen epithelial gene modules associated with ruminal pH and metabolites. A: WGCNA of rumen epithelial transcriptome correlation with ruminal pH and metabolites. B: Top 30 GO pathways of genes significantly in the MEblue module. C: Top 30 GO pathways of genes significantly in the MEgreen module. CON, nutritionally enriched group; LN, solid diet deficiency group received only milk.

Through transcriptome sequencing, a total of 408 776 226 high-quality reads were obtained from 16 samples (25 548 514 ± 3 470 260 reads per sample). We performed a comprehensive analysis of the DEGs in the rumen epithelial cells of the two groups, identifying a total of 20 496 genes. The PCoA revealed distinguishable clustering patterns, indicating that solid diet deficiency induced significant changes in gene expression within the rumen epithelial cells (Supplementary Fig. S8A). We observed 3023 DEGs [FDR < 0.05; |log2(FC)| > 1.5], comprising 772 significantly downregulated genes and 2251 significantly upregulated genes (Supplementary Fig. S8B). Utilizing weighted gene co-expression network analysis (WGCNA), key phenotype-related modules were identified through correlation pattern recognition, and the genes in rumen epithelial cells were systematically divided into 8 different modules. The MEblue module (comprising 10 389 genes) and MEgreen module (comprising 1174 genes) were found to have strong correlations with nitrogenous compounds and rumen fermentation parameters (Fig. 4A). By displaying the Top 30 enriched pathways in the MEblue module (Fig. 4B) and MEgreen module (Fig. 4C), associations with organelles, particularly mitochondria, were observed. This suggests that the lack of solid diet may lead to a disruption in the metabolic function of epithelial cells and an imbalance in cellular mitochondrial energy metabolism.

**Figure 5:**
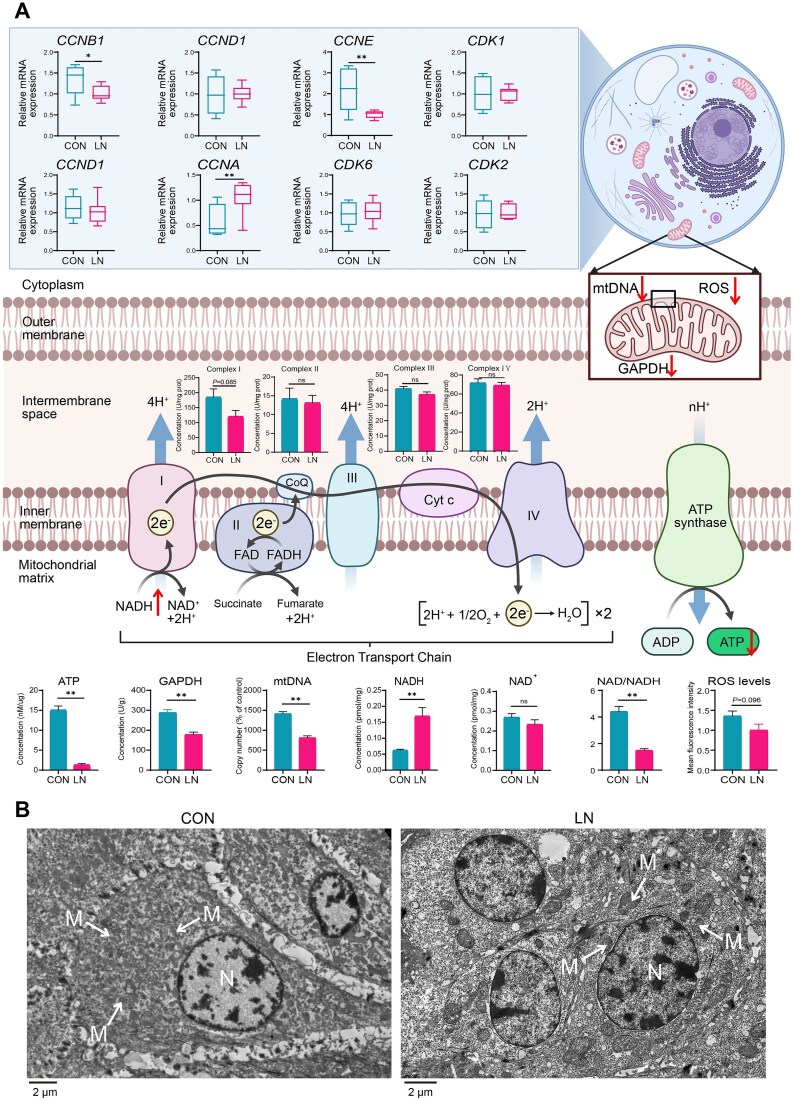
Overview of rumen epithelial cell differentiation and mitochondrial energy metabolism. A: Changes of rumen epithelial differentiation and mitochondrial energy metabolism. B: Scanning electron microscopy observation of epithelial cells. “M” represents mitochondrion, “N” represents nucleus. “*” represents *P* < .05, “**” represents *P* < .01; CON, nutritionally enriched group; LN, solid diet deficiency group received only milk. The data are presented as the mean ± SEM.

We further analyzed genes and proteins related to cell division and differentiation in the rumen epithelia (Fig. 5A). Specifically, to adapt to the energy deficiency caused by the absence of solid diet, rumen epithelial cells slowed down cell cycle progression by inhibiting the expression of genes *CCNB1* and *CCNE*. At the same time, they upregulated *CCNA* to allow more time for DNA replication and cell division, thereby enabling cells to use resources more efficiently and ensuring the integrity of genetic information. Moreover, mitochondrial function in epithelial cells was also impaired, as indicated by reduced ATP production, lower mtDNA copy number, decreased NAD/NADH levels, and a downward trend in complex I of the electron transport chain and ROS concentration. Scanning electron microscopy revealed morphological changes in both epithelial cell populations, clearly showing that mitochondria were sparse with abnormal shape and size under the absence of solid diet (Fig. 5B). Overall, the reduced energy supply from the rumen microbiota to the host leads to impaired differentiation of epithelial cells and obstructed mitochondrial energy metabolism.

## Discussion

The structure and function of rumen microbiota are shaped by their nutritional environment [41]. The decreased bacterial abundance and increased archaeal abundance observed under the absence of solid diet are also a result of this environmental influence [42]. Under extreme environmental or nutritional conditions, microbial populations in the gastrointestinal tract adapt to maintain their own survival by modulating energy redistribution through changes in their structure and composition, as previously reported [43]. Similarly, in situations of nutrient scarcity such as during animal hibernation, hosts utilize microbial metabolic plasticity to sustain survival during food shortages in winter by altering nitrogen recycling to provide energy for both the microbiota and the host [44]. This may also be a form of self-redemption for rumen microbiota survival in this study. The early establishment and customization of key microbiota within the rumen likely hold a pivotal role, as they occupy advantageous ecological niches and foster complex microbial networks with other bacteria. These early interactions can have lasting and profound effects on the host’s rumen fermentation, energy metabolism, and overall growth performance [45]. Furthermore, complex microbial networks have the potential to encompass a wider array of functions and establish a more resilient microbial environment [46]. The differences in core microbiota also imply a shift in the functional patterns of the rumen, with a decrease in microbial network strength under solid diet deficiency conditions, stemming from variations in the development of microbial communities under specific nutritional conditions.

Ruminal microbial fermentation of food directly influences the ruminal VFA concentrations [47]. The absence of solid diet in the rumen causes a severe shortage of available substrates, leading to a significant reduction in the ruminal VFA level [48]. This change, characterized by an energy substrate deficiency, reduces the output of microbial energy products to the host, leading to an extreme nutritional rumen micro-ecology. Microorganisms tend to utilize urea and ammonia as their preferred nutritional sources under extreme nutritional or environmental conditions, aiming to achieve self-replication and release of metabolic by-products [49]. This indirectly reflects the adaptive changes in the microbial nutritional network and the efficiency of nutrient transfer and utilization under extreme conditions [50, 51]. This also confirms our observation of more energy metabolism and nitrogen-related microorganisms being correlated with MCP, NH_3_-N, and urea. Absolutely, within the cohort of bacteria primarily engaged in protein digestion, multiple species like *Selenomonas* [52], *Fusobacterium* [53], *Lactobacillus* [54], *Lachnospira* [55], and *Succinivibrio* [56] are recognized for their production of protein-degrading enzymes. These microorganisms play a crucial role in the breakdown of proteins into valuable products, and their dilution suggests a potential decrease in the ability of rumen bacteria to metabolize proteins and amino acids. Like previous study [57], this pattern mirrors a potential weakening of rumen energy metabolism, likely stemming from reduced bacterial abundance. The lack of solid diet in the rumen might contribute to the colonization of active complex carbohydrate eukaryote and methanogenic archaea exhibiting methyl-coenzyme M reductase activity [58]. The increase in pyrimidine metabolism and purine metabolism could potentially be linked to changes in eukaryota abundance [59]. Under nutrient limitations, microorganisms invest additional vigor in producing various extracellular enzymes to acquire nutrients and energy. This phenomenon reflects the microbial characteristic of “exploitation competition” [3, 60].

The evolution of chemical ecological niches within microbial systems has the potential to enhance overall productivity and promote coexistence among microbial communities [61]. In this study, methenamine and L-carnitine respectively inhibit bacterial growth and MCP production [62–64], potentially leading to increased hydrogen production for archaeal ATP synthesis to support microorganisms, and residual ammonia serving as a direct energy source for microorganisms. At the same time, the enhanced synthesis of glutamine and glutamate within microbial organisms also reflects an improvement in efficiency in utilizing nitrogen sources. This increased efficiency supports ATP production for microbial replication and growth by enhancing the conversion efficiency of ammonia, particularly represented by strains like *Prevotella ruminicola* 23 and *Ruminococcus albus* 8 in the rumen. They exemplify how microorganisms ingeniously utilize nitrogen to generate energy for themselves [65–67]. Previous reports have indicated that in nutrient-poor environments, microorganisms employ “cheating” strategies to utilize the products secreted by others, losing or reducing their own secretions, and even directly using DNA as a nitrogen source to sustain their survival [40, 68]. Additionally, nutrient-poor environments select microorganisms with high nutrient utilization efficiency [69], which may corroborate the enhanced role of the glutamine and glutamate cycles. This illustrates how the gastrointestinal microbiota sustains its survival in extreme environments by optimizing nitrogen metabolism strategies.

Changes in substances secreted by microorganisms, such as glycosyltransferases, can cause cascading effects within epithelial cells [37]. Additionally, the interactions between the rumen microbiota and epithelial cells highlight the complex interplay that influences gene expression patterns and the function of rumen epithelial cells [70]. The regulation of *CCNB1*, *CCNE*, and *CCNA* in epithelial cells reflects a delayed cell cycle due to the limited energy supply from the rumen to the host. This results in reduced cellular metabolic capacity, impacting normal mitosis and DNA replication in epithelial cells [71–74]. This imbalance could result in chromosomal instability, thereby affecting genomic integrity [75]. This could potentially represent a self-protective mechanism adopted by cells to maintain genomic stability [76]. The slowing of the life replication cycle of epithelial cells is accompanied by the inhibition of mitochondrial energy transfer [77]. Mitochondrial complex I stands as the primary entry point for electrons into the electron transport chain, acting as the gateway for oxidative phosphorylation to generate ATP [78]. Diminished levels of mitochondrial complex I suggest disturbances in the electron transport chain [79]. Although there may be higher levels of NADH under solid diet deficiency conditions, the resulting NAD^+^ levels do not show significant differences. This suggests that the decrease in mitochondrial complex I affects the efficiency of electron transport, leading to a notable decline in ATP production. Additionally, the reduction of mtDNA also signifies a decrease in the energy production capacity of epithelial cells [80, 81]. Indeed, considering the morphology and quantity of mitochondria, it is evident that the efficiency of energy transport in the rumen epithelium has decreased. This further suggests that the lack of solid diet reduces external energy availability in the rumen, leading to epithelial cell mitochondrial dysfunction.

In conclusion, this study explored the adaptive survival strategies of rumen microbiota with solid diet deficiency. These strategies reshaped the distribution and energy metabolism within the ruminal microbial community, leading to a decrease in bacterial abundance and the Simpson Index, and simplifying the interaction network between microorganisms. Additionally, the enriched archaea and eukaryotes promoted the production of ATP and ammonia, respectively, supplying energy and nitrogen sources for microbial survival, while enhancing the intracellular glutamate-glutamine pathway. However, this self-serving adaptive strategy of the microbiota downregulated epithelial cell cycle proteins (*CCNB1*, *CCNE*), along with reducing mitochondrial DNA (mtDNA) and GAPDH levels, significantly diminishing cellular ATP production and hindering host development. These findings emphasize that early-life solid diet deprivation may compromise the health of mammals.

### Limitations and future directions

One limitation of our study is that, although we demonstrated the survival patterns and mechanisms of microorganisms under solid diet deficiency conditions, we cannot directly elucidate the specific pathways of hydrogen flux in the gastrointestinal tract under such conditions, nor can we detail the enhanced energy utilization patterns in microorganisms. The energy metabolism strategy of gastrointestinal microbiota and the resource allocation between its environmental characteristics have been a mysterious area of intense investigation, and future efforts should focus on quantifying the contribution of microbial competition to microbial community reshaping and metabolic efficiency, uncovering overall and comprehensive adaptive changes in the host, and utilizing multi-omics techniques and finer direct observations, such as metatranscriptome [82] and single-cell RNA sequencing technologies [83]. These methods will enable accurate prediction of the real responses of microbial communities and their adaptive processes in the context of competitive dynamics. Delving deeper into this mechanism offers valuable insights into the survival strategies of gastrointestinal microorganisms under extreme conditions, providing a reference for future studies on mammalian intestinal microecology.

## Supplementary Material

Revised_Supplementary_material_wraf064

## Data Availability

The raw sequence data from metagenomic assemblies and filtering of the rumen contents microbial community have been stored in the Genome Sequence Archive database of the China National Center for Bioinformation/Beijing Institute of Genomics, Chinese Academy of Sciences under accession number CRA014962 (https://ngdc.cncb.ac.cn/gsa/browse/CRA014962). The metabolomic datasets analyzed in this study have been archived in the OMIX database at the China National Center for Bioinformation/Beijing Institute of Genomics, Chinese Academy of Sciences, with the accession number OMIX005843 (https://ngdc.cncb.ac.cn/omix/release/OMIX005843). The raw transcriptomic data from rumen epithelial cells have been stored in the NCBI Sequence Read Archive under project number PRJNA881929 (https://www.ncbi.nlm.nih.gov/bioproject/PRJNA1208776/). Additionally, the 16S rRNA sequencing data are available at the National Center for Biotechnology Information under accession numbers PRJNA1209413 (http://www.ncbi.nlm.nih.gov/bioproject/1209413) and PRJNA1209636 (http://www.ncbi.nlm.nih.gov/bioproject/1209636).
