# Supplementary material for: Adaptive survival strategies of rumen microbiota with solid diet deficiency in early life cause epithelial mitochondrial dysfunction
Source: ISME J. 2025 Apr 6;19(1):wraf064. doi: 10.1093/ismejo/wraf064 (PMC12021266; doi:10.1093/ismejo/wraf064)
Supplement: Revised_Supplementary_material_wraf064 [file revised_supplementary_material_wraf064.docx]

**Table S1** Ingredient and chemical composition of the diet (dry matter basis).

| Item | Alfalfa hay | Corn-soybean starter |
| --- | --- | --- |
| Ingredients, % DM | | |
| Alfalfa hay | 98.50 | 0.00 |
| Corn | 0.00 | 56.00 |
| Soybean meal | 0.00 | 31.00 |
| Wheat | 0.00 | 4.00 |
| Whey powder | 0.00 | 5.00 |
| limestone meal powder | 0.00 | 1.00 |
| Calcium monophosphate | 0.00 | 1.50 |
| NaCl | 0.50 | 0.50 |
| Premix1 | 1.00 | 1.00 |
| Nutrient composition | | |
| Metabolic energy, MJ/kg DM2 | 9.69 | 12.67 |
| Crude protein, % DM | 18.76 | 19.62 |
| Ether extract, % DM | 2.26 | 3.68 |
| Crude fiber, % DM | 22.29 | 3.35 |
| Crude ash, % DM | 7.46 | 3.16 |
| NDF, % DM | 36.45 | 10.23 |
| ADF, % DM | 30.54 | 5.11 |
| Ca, % DM | 1.38 | 1.04 |
| P, % DM | 0.50 | 0.50 |

^1^ Contained 102g·kg-1 of Zn，47g·kg-1 of Mn，26g· kg-1 of Cu，1140 mg·kg-1 of I，500 mg·kg-1 of Se，340 mg·kg-1 of Co，17167380IU·kg-1 of vitamin A，858370IU·kg-1 of vitamin D，and 23605IU·kg-1 of vitamin E.

^2^ Calculated based on ministry of agriculture of china recommendations (MOA, 2004). DM, dry matter.

**Table S2** Overview of animal performance of lambs during the trial period.

| Item | CON | LN | *P*-value |
| --- | --- | --- | --- |
| Initial body weight, kg | 5.81 ± 0.33 | 5.95 ± 0.21 | 0.753 |
| Final body weight, kg | 11.13 ± 1.71 | 9.80 ± 0.99 | 0.079 |
| Weight gain, kg | 5.31 ± 0.34 | 3.86 ± 0.19 | 0.002 |
| Average daily weight gain, g | 196.99 ± 12.59 | 142.87 ± 7.10 | 0.002 |
| Average daily solid diet intake, g | 263.59 ± 36.35 | - | - |
| Average daily milk intake, mL | 600.00 ± 0.00 | 1830.53 ± 92.41 | - |

The values are shown as means±SEM; *P* < 0.05 indicated that mean values were significantly different. CON, nutritionally enriched group; LN, solid diet deficiency group received only milk.

**Table S3** Primers sequence used in the present study.

| Gene | Gene ID | Primer sequence (5’→3’) | Amplicon size, bp |
| --- | --- | --- | --- |
| *CCNA* | NC_019478.2 | F: CTCTCCTATCACCGCCTGAC  R: CTTTGGGGTCCAAGTTCTGC | 142 |
| *CCNB1* | NM_001045872.1 | F: AGCGGATCCAAACCTTTGTAGTG  R: CAATGAGGATGGCTCTCATGTTTC | 137 |
| *CCND1* | NC_019478.2 | F: CGATAAGCCAGCTAACGGGG  R: CAGGCTGCCTCCGTCTG | 103 |
| *CCNE* | XM_015100542 | F: TGGCACCGATGTCTCTGTTC  R: CCACACTGGCTTCTCACAGT | 114 |
| *CDK1* | NM_174016.2 | F: CCAATAATGAAGTGTGGCCAGAAG  R: AGAAATTCGTTTGGCAGGATCATAG | 164 |
| *CDK2* | NM_001142509.1 | F: CCTAGCTTTCTGCCACTCTCAT  R: TCACCACCTCGTGGGTATAAGT | 153 |
| *CDK4* | NM_001127269.1 | F: GACCAAGACCTCAGGACGTATC  R: CACCACTTGTCACCAGAATGTT | 250 |
| *CDK6* | XM_012177413.2 | F: GATGGCTCTTACCTCAGTGGTT  R: GGGTAGGGCAACATCTCTAGG | 228 |
| *GAPDH* | NM_001190390.1 | F: GGGTCATCATCTCTGCACCT  R: GGTCATAAGTCCCTCCACGA | 180 |

**Table S4** Differential metabolites in the rumen.

| Name | Log2(FC) | VIP | FDR  Adjusted *P* value |
| --- | --- | --- | --- |
| 3-Deoxyguanosine | -17.89 | 1.28 | ＜0.01 |
| 2'-Deoxyuridine | -11.49 | 1.49 | ＜0.01 |
| 9,10,13-Trihydroxystearic acid | -7.09 | 1.54 | ＜0.01 |
| Deoxyinosine | -6.97 | 1.32 | ＜0.01 |
| N-Acetylcadaverine | -4.83 | 1.12 | ＜0.01 |
| 12(13)-EpOME | -4.81 | 1.31 | ＜0.01 |
| Daidzein | -4.67 | 1.05 | ＜0.01 |
| 1-Aminocyclohexanecarboxylic acid | -4.51 | 1.19 | ＜0.01 |
| Deoxyadenosine | -4.15 | 1.07 | ＜0.01 |
| Enterolactone | -4.06 | 1.28 | ＜0.01 |
| resorcinol | -3.96 | 1.45 | 0.002 |
| 3-Methyldioxyindole | -3.91 | 1.47 | ＜0.01 |
| 9,10-DiHODE | -3.87 | 1.28 | 0.03 |
| p-Salicylic acid | -3.82 | 1.25 | ＜0.01 |
| 8,9-EE-14(Z)-E | -3.77 | 1.28 | ＜0.01 |
| Traumatic Acid | -3.76 | 1.31 | 0.02 |
| O-Desmethylangolensin | -3.61 | 1.10 | 0.002 |
| 9,10,13-TriHOME | -3.44 | 1.48 | ＜0.01 |
| Ricinoleic acid | -3.29 | 1.19 | ＜0.01 |
| Oxyphenonium | -3.24 | 1.47 | ＜0.01 |
| 4-Pyridoxic acid | -3.05 | 1.42 | ＜0.01 |
| 2-Indolecarboxylic acid | -2.99 | 1.54 | ＜0.01 |
| Xanthine | -2.84 | 1.39 | ＜0.01 |
| 3,3-Dimethylglutaric acid | -2.77 | 1.02 | 0.03 |
| Butyric acid | -2.63 | 1.34 | ＜0.01 |
| Apigenin | -2.62 | 1.03 | 0.05 |
| N-ACETYLPROLINE | -2.59 | 1.19 | ＜0.01 |
| Hypoxanthine | -2.47 | 1.32 | ＜0.01 |
| Guanine | -2.42 | 1.36 | ＜0.01 |
| 12,13-DiHOME | -2.40 | 1.30 | ＜0.01 |
| 5-Hydroxyindoleacetic acid | -2.31 | 1.45 | ＜0.01 |
| Indole-3-carboxaldehyde | -2.19 | 1.43 | ＜0.01 |
| Linoleic acid | -2.17 | 1.34 | ＜0.01 |
| Gentisic acid | -1.94 | 1.33 | ＜0.01 |
| 3-ketosphinganine | -1.94 | 1.33 | ＜0.01 |
| (2'E,4'Z,7'Z,8E)-Colnelenic acid | -1.73 | 1.39 | ＜0.01 |
| Nandrolone | -1.64 | 1.43 | ＜0.01 |
| Corey PG-Lactone Diol | -1.55 | 1.44 | ＜0.01 |
| 2-hydroxyhexadecanoic acid | -1.55 | 1.08 | ＜0.01 |
| Dodecanedioic acid | -1.53 | 1.08 | ＜0.01 |
| Sphingosine | 1.57 | 1.33 | ＜0.01 |
| PE(P-16:0e/0:0) | 1.62 | 1.20 | ＜0.01 |
| Oxprenolol | 1.74 | 1.38 | ＜0.01 |
| Uridine | 1.87 | 1.36 | ＜0.01 |
| L-Leucine | 1.87 | 1.03 | ＜0.01 |
| N-palmitoyl serine | 2.07 | 1.42 | ＜0.01 |
| 3-Hydroxypicolinic acid | 2.08 | 1.45 | ＜0.01 |
| L-Phenylalanine | 2.08 | 1.09 | ＜0.01 |
| Glutaric acid | 2.12 | 1.07 | ＜0.01 |
| L-Tryptophan | 2.12 | 1.31 | ＜0.01 |
| L-Proline | 2.12 | 1.14 | ＜0.01 |
| Bis(2-ethylhexyl) phthalate | 2.14 | 1.37 | ＜0.01 |
| Phe Pro Ile | 2.18 | 1.02 | ＜0.01 |
| m-Xylene | 2.18 | 1.42 | ＜0.01 |
| 2,5-Dimethylbenzaldehyde | 2.20 | 1.41 | ＜0.01 |
| Monoethylhexyl phthalic acid | 2.30 | 1.47 | ＜0.01 |
| N-Acetyl-L-glutamic acid | 2.30 | 1.36 | ＜0.01 |
| Phytosphingosine | 2.33 | 1.46 | ＜0.01 |
| 4-Hydroxy-2,6,6-trimethyl-3-oxo-1,4-cyclohexadiene-1-carboxaldehyde | 2.33 | 1.43 | ＜0.01 |
| D-Lactic acid | 2.34 | 1.08 | ＜0.01 |
| 1-Monopalmitin | 2.39 | 1.38 | ＜0.01 |
| Sphinganine | 2.41 | 1.44 | ＜0.01 |
| Methenamine | 2.45 | 1.43 | ＜0.01 |
| Nervonyl carnitine | 2.53 | 1.44 | ＜0.01 |
| 2-(3-Phenylpropyl)tetrahydrofuran | 2.53 | 1.51 | ＜0.01 |
| L-Glutamate | 2.54 | 1.13 | ＜0.01 |
| 13Z-Docosenamide | 2.69 | 1.11 | 0.02 |
| Oleoyl Ethyl Amide | 2.87 | 1.27 | ＜0.01 |
| TG(18:0/18:2(9Z,12Z)/20:5(5Z,8Z,11Z,14Z,17Z)) | 2.87 | 1.45 | ＜0.01 |
| Citrulline | 2.91 | 1.28 | ＜0.01 |
| beta-Alaninebetaine | 2.92 | 1.33 | ＜0.01 |
| Pro Val | 3.13 | 1.17 | ＜0.01 |
| TG(16:0/18:1(9Z)/20:5(5Z,8Z,11Z,14Z,17Z))[iso6] | 3.13 | 1.45 | ＜0.01 |
| Pro Pro | 3.88 | 1.17 | ＜0.01 |
| 9,12,15-Octadecatrien-1-ol | 3.99 | 1.22 | ＜0.01 |
| L-Carnitine | 4.81 | 1.43 | ＜0.01 |
| Dimethylethanolamine | 6.13 | 1.10 | ＜0.01 |

**Table S5** Correlation information of microbial network nodes in LN and CON groups.

| LN | | | | CON | | | |
| --- | --- | --- | --- | --- | --- | --- | --- |
| ID | degree | closeness Centrality | betweenness Centrality | ID | degree | closeness Centrality | betweenness Centrality |
| *Bacteroides* | 2 | 26.34 | 0.00 | *Kandleria* | 3 | 31.08 | 0.00 |
| *Fibrobacter* | 2 | 28.54 | 0.00 | *Desulfobulbus* | 2 | 30.12 | 0.00 |
| *Mediterranea* | 2 | 23.76 | 0.00 | *Muribaculum* | 3 | 30.03 | 0.00 |
| *Desulfovibrio* | 1 | 1.00 | 0.00 | *Flavonifractor* | 1 | 26.48 | 0.00 |
| *Proteiniphilum* | 1 | 1.00 | 0.00 | *Streptococcus* | 1 | 24.93 | 0.00 |
| *Chlamydia* | 1 | 22.95 | 0.00 | *Butyricimonas* | 2 | 24.23 | 1.00 |
| *Fusobacterium* | 1 | 21.93 | 0.00 | *Tannerella* | 3 | 28.98 | 2.34 |
| *Megasphaera* | 2 | 25.13 | 0.67 | *Megamonas* | 2 | 24.57 | 2.59 |
| *Odoribacter* | 2 | 32.52 | 6.78 | *Methanosphaera* | 8 | 39.47 | 3.47 |
| *Blautia* | 3 | 32.20 | 8.31 | *Anaerotruncus* | 4 | 36.72 | 4.22 |
| *Kiritimatiella* | 5 | 35.12 | 9.02 | *Acidaminococcus* | 9 | 42.80 | 6.02 |
| *Selenomonas* | 3 | 30.56 | 10.25 | *Desulfovibrio* | 4 | 35.92 | 7.05 |
| *Faecalibacterium* | 14 | 43.78 | 13.04 | *Roseburia* | 5 | 36.45 | 9.80 |
| *Muribaculum* | 2 | 27.93 | 17.82 | *Pyramidobacter* | 4 | 34.35 | 20.02 |
| *Oscillibacter* | 11 | 41.25 | 19.43 | *Faecalibacterium* | 13 | 43.72 | 24.90 |
| *Pyramidobacter* | 3 | 26.46 | 26.10 | *Agathobacter* | 2 | 29.58 | 25.45 |
| *Olsenella* | 5 | 34.87 | 26.33 | *Barnesiella* | 4 | 31.70 | 30.14 |
| *Luteimonas* | 4 | 32.58 | 34.21 | *Clostridium* | 4 | 36.40 | 36.07 |
| *Elusimicrobium* | 4 | 33.32 | 39.93 | *Paenibacillus* | 8 | 40.68 | 42.85 |
| *Paenibacillus* | 12 | 40.75 | 43.02 | *Blautia* | 11 | 40.92 | 46.98 |
| *Butyricicoccus* | 4 | 30.92 | 51.75 | *Subdoligranulum* | 3 | 34.75 | 49.39 |
| *Acholeplasma* | 4 | 31.48 | 52.37 | *Phocaeicola* | 5 | 35.13 | 51.18 |
| *Neglecta* | 18 | 46.25 | 52.81 | *Odoribacter* | 5 | 34.37 | 59.13 |
| *Victivallis* | 5 | 35.15 | 53.10 | *Clostridioides* | 12 | 44.52 | 59.42 |
| *Oribacterium* | 6 | 36.90 | 58.62 | *Caecibacter* | 3 | 32.02 | 64.01 |
| *Alloprevotella* | 5 | 32.98 | 58.76 | *Treponema* | 3 | 35.08 | 67.09 |
| *Petrimonas* | 3 | 31.88 | 62.58 | *Pseudoscardovia* | 5 | 36.93 | 72.16 |
| *Anaerocolumna* | 4 | 31.20 | 66.48 | *Fusobacterium* | 13 | 44.77 | 81.91 |
| *Succiniclasticum* | 3 | 29.70 | 66.96 | *Pseudoramibacter* | 3 | 31.15 | 84.64 |
| *Lentimicrobium* | 4 | 35.87 | 67.25 | *Dorea* | 19 | 49.40 | 91.36 |
| *Paludibacter* | 3 | 31.13 | 67.42 | *Allisonella* | 15 | 48.22 | 92.08 |
| *Coprobacillus* | 7 | 38.85 | 74.41 | *Pseudobutyrivibrio* | 10 | 42.02 | 98.49 |
| *Phocaeicola* | 5 | 35.05 | 80.28 | *Tolumonas* | 4 | 36.32 | 103.69 |
| *Methanobrevibacter* | 4 | 30.68 | 86.21 | *Succinatimonas* | 17 | 47.02 | 112.17 |
| *Slackia* | 18 | 46.33 | 86.68 | *Succinivibrio* | 17 | 47.02 | 112.17 |
| *Coprobacter* | 6 | 35.17 | 92.90 | *Lactomassilus* | 4 | 37.28 | 112.37 |
| *Intestinimonas* | 14 | 42.60 | 96.08 | *Oribacterium* | 11 | 45.47 | 126.94 |
| *Mycoplasma* | 14 | 42.60 | 96.08 | *Olsenella* | 5 | 34.93 | 128.34 |
| *Paraprevotella* | 9 | 41.65 | 98.39 | *Drancourtella* | 21 | 51.73 | 131.71 |
| *Prevotella* | 5 | 36.18 | 101.29 | *Faecalicatena* | 17 | 49.57 | 131.91 |
| *Treponema* | 5 | 31.98 | 110.33 | *Dialister* | 15 | 48.30 | 132.76 |
| *Lachnoclostridium* | 14 | 42.63 | 113.04 | *Veillonella* | 11 | 44.07 | 136.40 |
| *Barnesiella* | 5 | 32.82 | 115.17 | *Ruminobacter* | 6 | 39.02 | 136.63 |
| *Azoarcus* | 7 | 36.85 | 116.68 | *Sharpea* | 17 | 47.90 | 138.14 |
| *Thauera* | 7 | 36.85 | 116.68 | *Mitsuokella* | 8 | 40.78 | 147.58 |
| *Dysgonomonas* | 4 | 33.70 | 124.10 | *Sphingomonas* | 19 | 49.65 | 148.89 |
| *Bilophila* | 4 | 33.40 | 125.02 | *Eubacterium* | 7 | 41.62 | 149.06 |
| *Butyrivibrio* | 19 | 47.83 | 125.51 | *Hungatella* | 9 | 42.27 | 159.64 |
| *Hungatella* | 19 | 47.83 | 125.51 | *Massiliomicrobiota* | 8 | 39.18 | 160.80 |
| *Alistipes* | 4 | 34.20 | 127.39 | *Prevotella* | 6 | 40.92 | 164.01 |
| *Ruthenibacterium* | 19 | 45.38 | 132.00 | *Anaerostipes* | 20 | 50.73 | 164.85 |
| *Endomicrobium* | 20 | 46.67 | 144.70 | *Campylobacter* | 5 | 38.12 | 165.41 |
| *Syntrophomonas* | 4 | 33.13 | 163.85 | *Acholeplasma* | 13 | 45.98 | 174.69 |
| *Capnocytophaga* | 6 | 35.78 | 175.89 | *Alistipes* | 17 | 49.32 | 176.20 |
| *Porphyromonas* | 6 | 37.77 | 184.98 | *Mediterranea* | 4 | 35.32 | 177.03 |
| *Anaeromassilibacillus* | 20 | 48.38 | 186.38 | *Propionispira* | 9 | 41.33 | 182.36 |
| *Prevotellamassilia* | 8 | 34.72 | 190.09 | *Eisenbergiella* | 19 | 50.32 | 186.02 |
| *Dorea* | 21 | 49.63 | 190.45 | *Solobacterium* | 9 | 42.23 | 187.83 |
| *Cloacibacillus* | 4 | 30.62 | 192.00 | *Bacteroides* | 5 | 32.68 | 189.80 |
| *Candidatus Symbiothrix* | 6 | 37.43 | 195.58 | *Aeromonas* | 3 | 33.63 | 196.00 |
| *Actinomyces* | 11 | 41.63 | 206.80 | *Anaeromassilibacillus* | 18 | 49.82 | 199.93 |
| *Acidaminococcus* | 5 | 36.23 | 207.81 | *Butyricicoccus* | 18 | 49.90 | 205.55 |
| *Flavobacterium* | 9 | 40.67 | 210.15 | *Coprobacillus* | 8 | 42.18 | 208.39 |
| *Ruminococcus* | 21 | 49.52 | 218.35 | *Lachnoclostridium* | 21 | 51.23 | 208.45 |
| *Pseudoflavonifractor* | 21 | 48.05 | 223.76 | *Entodinium* | 12 | 44.83 | 212.65 |
| *Neisseria* | 6 | 35.78 | 227.15 | *Alloprevotella* | 7 | 38.97 | 212.99 |
| *Anaerovorax* | 4 | 29.07 | 233.26 | *Parabacteroides* | 5 | 31.77 | 221.02 |
| *Sporobacter* | 7 | 37.42 | 240.65 | *Fusicatenibacter* | 8 | 39.75 | 223.27 |
| *Corynebacterium* | 6 | 36.05 | 241.49 | *Acetivibrio* | 17 | 49.18 | 223.51 |
| *Sphaerochaeta* | 4 | 38.70 | 242.81 | *Porphyromonas* | 6 | 35.83 | 227.84 |
| *Bacillus* | 6 | 35.62 | 260.51 | *Anaerobiospirillum* | 7 | 41.30 | 230.10 |
| *Flexilinea* | 5 | 31.68 | 268.48 | *Butyrivibrio* | 17 | 49.52 | 235.78 |
| *Eubacterium* | 7 | 36.57 | 278.99 | *Selenomonas* | 5 | 33.48 | 241.55 |
| *Mobilibacterium* | 5 | 34.45 | 279.49 | *Coprococcus* | 14 | 46.85 | 254.30 |
| *Staphylococcus* | 6 | 36.30 | 290.53 | *Sphaerochaeta* | 6 | 32.57 | 256.40 |
| *Synergistes* | 4 | 33.88 | 293.74 | *Bifidobacterium* | 9 | 41.77 | 261.65 |
| *Acetatifactor* | 15 | 47.27 | 298.75 | *Succiniclasticum* | 18 | 50.73 | 270.01 |
| *Methanomicrobium* | 5 | 36.77 | 304.70 | *Lachnospira* | 12 | 44.95 | 271.71 |
| *Parabacteroides* | 10 | 39.90 | 307.41 | *Oscillibacter* | 26 | 54.40 | 275.42 |
| *Anaerotruncus* | 21 | 48.72 | 329.80 | *Mobilibacterium* | 8 | 40.47 | 280.13 |
| *Roseburia* | 13 | 46.45 | 343.46 | *Schwartzia-* | 6 | 35.58 | 281.25 |
| *Streptococcus* | 10 | 41.47 | 346.49 | *Ruminococcus* | 9 | 44.35 | 302.67 |
| *Coprococcus* | 15 | 47.17 | 348.71 | *Bacillus* | 14 | 48.15 | 307.71 |
| *Mogibacterium* | 10 | 42.93 | 352.85 | *Chlamydia* | 10 | 44.02 | 330.90 |
| *Bifidobacterium* | 9 | 41.62 | 360.78 | *Coprobacter* | 6 | 38.88 | 333.47 |
| *Gemmiger* | 17 | 46.88 | 375.66 | *Anaerovibrio* | 6 | 35.87 | 350.77 |
| *Flavonifractor* | 24 | 50.30 | 377.97 | *Methanobrevibacter* | 12 | 46.65 | 354.95 |
| *Hungateiclostridium* | 6 | 38.37 | 379.95 | *Sarcina* | 14 | 48.28 | 368.69 |
| *Lactobacillus* | 13 | 45.07 | 422.51 | *Phascolarctobacterium* | 9 | 45.03 | 368.73 |
| *Alysiella* | 6 | 31.77 | 426.25 | *Megasphaera* | 17 | 49.82 | 382.37 |
| *Campylobacter* | 7 | 38.12 | 448.20 | *Fibrobacter* | 8 | 40.92 | 385.06 |
| *Sarcina* | 6 | 39.30 | 451.37 | *Paraprevotella* | 15 | 48.82 | 423.11 |
| *Pseudobutyrivibrio* | 13 | 43.78 | 502.73 | *Suttonella* | 6 | 42.33 | 429.04 |
| *Methanosarcina* | 8 | 38.93 | 510.41 | *Stomatobaculum* | 11 | 44.10 | 431.85 |
| *Subdoligranulum* | 19 | 49.43 | 595.03 | *Shuttleworthia* | 7 | 37.30 | 442.23 |
| *Desulfobulbus* | 15 | 47.62 | 624.92 | *Lactobacillus* | 8 | 40.70 | 485.70 |
| *Clostridium* | 20 | 51.00 | 646.19 | *Mycoplasma* | 21 | 52.77 | 536.52 |
| *Akkermansia* | 10 | 44.43 | 817.28 | *Prevotellamassilia* | 6 | 41.20 | 561.11 |
| *Tannerella* | 9 | 41.65 | 832.51 | *Succinimonas* | 11 | 44.92 | 677.57 |
| *Butyricimonas* | 13 | 45.25 | 1105.46 | *Marvinbryantia* | 27 | 57.17 | 986.46 |

**Table S6** Differences in carbohydrate enzyme family level.

| Class | Family | CON | LN | *P* value |
| --- | --- | --- | --- | --- |
| AA | AA10 | 0.000 ± 0.000 | 0.021 ± 0.018 | 0.016 |
|  | AA12 | 0.000 ± 0.000 | 0.003 ± 0.001 | 0.003 |
|  | AA2 | 0.001 ± 0.000 | 0.004 ± 0.001 | 0.001 |
|  | AA4 | 0.312 ± 0.060 | 0.078 ± 0.014 | 0.001 |
|  | AA6 | 1.743 ± 0.110 | 2.215 ± 0.107 | 0.015 |
|  | AA7 | 0.098 ± 0.020 | 0.030 ± 0.003 | ＜0.001 |
| CBM | CBM10 | 0.000 ± 0.000 | 0.001 ± 0.000 | 0.024 |
|  | CBM14 | 0.000 ± 0.000 | 0.002 ± 0.001 | 0.022 |
|  | CBM16 | 0.070 ± 0.021 | 0.435 ± 0.052 | ＜0.001 |
|  | CBM19 | 0.000 ± 0.000 | 0.001 ± 0.001 | 0.038 |
|  | CBM20 | 0.729 ± 0.056 | 0.392 ± 0.046 | 0.002 |
|  | CBM25 | 0.172 ± 0.035 | 0.014 ± 0.003 | ＜0.001 |
|  | CBM26 | 1.277 ± 0.181 | 0.196 ± 0.039 | ＜0.001 |
|  | CBM27 | 0.012 ± 0.004 | 0.003 ± 0.001 | 0.028 |
|  | CBM29 | 0.000 ± 0.000 | 0.001 ± 0.000 | 0.009 |
|  | CBM32 | 1.245 ± 0.065 | 2.769 ± 0.133 | ＜0.001 |
|  | CBM34 | 0.484 ± 0.064 | 0.124 ± 0.017 | ＜0.001 |
|  | CBM37 | 0.506 ± 0.126 | 0.754 ± 0.073 | 0.021 |
|  | CBM38 | 0.001 ± 0.000 | 0.049 ± 0.014 | ＜0.001 |
|  | CBM39 | 0.001 ± 0.001 | 0.007 ± 0.002 | 0.003 |
|  | CBM40 | 0.098 ± 0.019 | 0.243 ± 0.018 | 0.001 |
|  | CBM42 | 0.035 ± 0.008 | 0.001 ± 0.000 | ＜0.001 |
|  | CBM44 | 0.168 ± 0.019 | 0.727 ± 0.107 | ＜0.001 |
|  | CBM46 | 0.095 ± 0.014 | 0.023 ± 0.004 | 0.001 |
|  | CBM47 | 0.007 ± 0.005 | 0.039 ± 0.019 | 0.015 |
|  | CBM5 | 0.076 ± 0.027 | 0.015 ± 0.002 | 0.001 |
|  | CBM50 | 1.813 ± 0.116 | 2.594 ± 0.173 | 0.007 |
|  | CBM51 | 0.032 ± 0.009 | 0.127 ± 0.038 | 0.002 |
|  | CBM52 | 0.000 ± 0.000 | 0.002 ± 0.001 | 0.007 |
|  | CBM55 | 0.000 ± 0.000 | 0.001 ± 0.000 | 0.010 |
|  | CBM56 | 0.229 ± 0.032 | 0.956 ± 0.172 | ＜0.001 |
|  | CBM57 | 0.073 ± 0.012 | 0.020 ± 0.006 | 0.001 |
|  | CBM58 | 0.001 ± 0.001 | 0.012 ± 0.005 | 0.001 |
|  | CBM59 | 0.020 ± 0.004 | 0.002 ± 0.000 | ＜0.001 |
|  | CBM60 | 0.000 ± 0.000 | 0.002 ± 0.001 | 0.003 |
|  | CBM62 | 0.050 ± 0.014 | 0.085 ± 0.013 | 0.028 |
|  | CBM63 | 0.042 ± 0.009 | 0.006 ± 0.001 | ＜0.001 |
|  | CBM64 | 0.001 ± 0.001 | 0.007 ± 0.001 | 0.002 |
|  | CBM66 | 0.170 ± 0.025 | 0.312 ± 0.032 | 0.005 |
|  | CBM67 | 0.724 ± 0.017 | 0.982 ± 0.084 | 0.015 |
|  | CBM70 | 0.001 ± 0.000 | 0.013 ± 0.001 | ＜0.001 |
|  | CBM72 | 0.064 ± 0.011 | 0.022 ± 0.005 | 0.005 |
|  | CBM74 | 0.075 ± 0.027 | 0.001 ± 0.000 | ＜0.001 |
|  | CBM76 | 0.013 ± 0.004 | 0.002 ± 0.001 | 0.021 |
|  | CBM77 | 0.107 ± 0.014 | 0.007 ± 0.002 | ＜0.001 |
|  | CBM78 | 0.003 ± 0.001 | 0.068 ± 0.011 | ＜0.001 |
|  | CBM79 | 0.001 ± 0.000 | 0.003 ± 0.001 | 0.010 |
|  | CBM9 | 0.111 ± 0.019 | 0.379 ± 0.028 | ＜0.001 |
| CE | CE1 | 2.955 ± 0.141 | 4.907 ± 0.297 | ＜0.001 |
|  | CE10 | 2.826 ± 0.093 | 3.705 ± 0.244 | 0.007 |
|  | CE11 | 0.432 ± 0.040 | 0.570 ± 0.038 | 0.038 |
|  | CE12 | 0.752 ± 0.069 | 0.453 ± 0.058 | 0.005 |
|  | CE14 | 0.263 ± 0.026 | 0.863 ± 0.087 | ＜0.001 |
|  | CE15 | 0.143 ± 0.020 | 0.195 ± 0.019 | 0.028 |
|  | CE16 | 0.000 ± 0.000 | 0.001 ± 0.000 | 0.002 |
|  | CE3 | 1.029 ± 0.068 | 1.353 ± 0.053 | 0.002 |
|  | CE4 | 1.772 ± 0.144 | 1.176 ± 0.051 | 0.001 |
|  | CE5 | 0.094 ± 0.008 | 0.038 ± 0.008 | 0.001 |
|  | CE8 | 0.598 ± 0.045 | 0.324 ± 0.045 | 0.001 |
|  | CE9 | 0.319 ± 0.038 | 0.454 ± 0.032 | 0.050 |
| GH | GH1 | 0.437 ± 0.065 | 0.103 ± 0.013 | ＜0.001 |
|  | GH102 | 0.002 ± 0.000 | 0.014 ± 0.003 | ＜0.001 |
|  | GH104 | 0.001 ± 0.001 | 0.008 ± 0.002 | 0.001 |
|  | GH105 | 0.550 ± 0.029 | 0.266 ± 0.047 | 0.001 |
|  | GH109 | 1.553 ± 0.125 | 2.802 ± 0.096 | ＜0.001 |
|  | GH11 | 0.008 ± 0.004 | 0.037 ± 0.007 | 0.002 |
|  | GH110 | 0.011 ± 0.003 | 0.154 ± 0.026 | ＜0.001 |
|  | GH115 | 0.351 ± 0.046 | 0.099 ± 0.020 | 0.001 |
|  | GH116 | 0.006 ± 0.002 | 0.035 ± 0.004 | ＜0.001 |
|  | GH117 | 0.003 ± 0.001 | 0.026 ± 0.006 | ＜0.001 |
|  | GH119 | 0.005 ± 0.005 | 0.002 ± 0.001 | 0.031 |
|  | GH121 | 0.015 ± 0.005 | 0.000 ± 0.000 | 0.010 |
|  | GH123 | 0.030 ± 0.006 | 0.159 ± 0.015 | ＜0.001 |
|  | GH125 | 0.109 ± 0.017 | 0.171 ± 0.020 | 0.038 |
|  | GH129 | 0.015 ± 0.006 | 0.068 ± 0.006 | 0.001 |
|  | GH13 | 4.973 ± 0.165 | 2.928 ± 0.199 | ＜0.001 |
|  | GH130 | 0.290 ± 0.014 | 0.339 ± 0.019 | 0.050 |
|  | GH131 | 0.000 ± 0.000 | 0.001 ± 0.001 | 0.013 |
|  | GH134 | 0.012 ± 0.006 | 0.000 ± 0.000 | ＜0.001 |
|  | GH15 | 0.058 ± 0.027 | 0.015 ± 0.003 | 0.050 |
|  | GH16 | 0.573 ± 0.047 | 0.928 ± 0.099 | 0.001 |
|  | GH17 | 0.000 ± 0.000 | 0.004 ± 0.001 | 0.003 |
|  | GH2 | 2.468 ± 0.134 | 3.008 ± 0.143 | 0.028 |
|  | GH20 | 0.441 ± 0.062 | 2.019 ± 0.140 | ＜0.001 |
|  | GH22 | 0.032 ± 0.008 | 0.000 ± 0.000 | ＜0.001 |
|  | GH23 | 1.120 ± 0.078 | 1.513 ± 0.084 | 0.005 |
|  | GH24 | 0.266 ± 0.041 | 0.047 ± 0.007 | ＜0.001 |
|  | GH25 | 1.405 ± 0.097 | 0.394 ± 0.036 | ＜0.001 |
|  | GH27 | 0.134 ± 0.018 | 0.368 ± 0.030 | ＜0.001 |
|  | GH28 | 1.238 ± 0.101 | 0.681 ± 0.084 | 0.002 |
|  | GH29 | 0.422 ± 0.046 | 1.410 ± 0.086 | ＜0.001 |
|  | GH32 | 0.918 ± 0.078 | 0.452 ± 0.072 | 0.001 |
|  | GH33 | 0.370 ± 0.018 | 1.106 ± 0.088 | ＜0.001 |
|  | GH35 | 0.339 ± 0.032 | 0.214 ± 0.021 | 0.007 |
|  | GH38 | 0.097 ± 0.021 | 0.198 ± 0.034 | 0.021 |
|  | GH39 | 0.067 ± 0.007 | 0.155 ± 0.015 | ＜0.001 |
|  | GH44 | 0.003 ± 0.001 | 0.006 ± 0.002 | 0.050 |
|  | GH45 | 0.003 ± 0.001 | 0.020 ± 0.005 | 0.001 |
|  | GH47 | 0.000 ± 0.000 | 0.010 ± 0.003 | 0.001 |
|  | GH50 | 0.014 ± 0.004 | 0.073 ± 0.022 | 0.001 |
|  | GH51 | 0.765 ± 0.052 | 0.449 ± 0.058 | 0.005 |
|  | GH53 | 0.683 ± 0.047 | 0.372 ± 0.054 | 0.002 |
|  | GH54 | 0.001 ± 0.000 | 0.009 ± 0.002 | ＜0.001 |
|  | GH55 | 0.051 ± 0.015 | 0.010 ± 0.001 | 0.015 |
|  | GH59 | 0.063 ± 0.012 | 0.025 ± 0.004 | 0.021 |
|  | GH63 | 0.065 ± 0.010 | 0.034 ± 0.006 | 0.028 |
|  | GH65 | 0.195 ± 0.027 | 0.091 ± 0.020 | 0.010 |
|  | GH67 | 0.151 ± 0.019 | 0.063 ± 0.013 | 0.002 |
|  | GH71 | 0.000 ± 0.000 | 0.001 ± 0.001 | 0.002 |
|  | GH73 | 1.432 ± 0.110 | 0.572 ± 0.028 | ＜0.001 |
|  | GH76 | 0.262 ± 0.015 | 0.141 ± 0.017 | 0.001 |
|  | GH77 | 0.864 ± 0.030 | 0.578 ± 0.036 | ＜0.001 |
|  | GH79 | 0.019 ± 0.007 | 0.001 ± 0.000 | 0.010 |
|  | GH8 | 0.128 ± 0.033 | 0.070 ± 0.014 | 0.050 |
|  | GH81 | 0.008 ± 0.007 | 0.003 ± 0.001 | 0.039 |
|  | GH85 | 0.019 ± 0.006 | 0.115 ± 0.028 | 0.001 |
|  | GH89 | 0.187 ± 0.013 | 0.482 ± 0.042 | ＜0.001 |
|  | GH9 | 0.223 ± 0.024 | 0.321 ± 0.030 | 0.021 |
|  | GH92 | 0.498 ± 0.025 | 1.385 ± 0.090 | ＜0.001 |
|  | GH93 | 0.026 ± 0.006 | 0.049 ± 0.006 | 0.021 |
|  | GH97 | 0.892 ± 0.054 | 1.182 ± 0.050 | 0.005 |
|  | GH98 | 0.044 ± 0.008 | 0.015 ± 0.004 | 0.010 |
| GT | GT10 | 0.068 ± 0.015 | 0.160 ± 0.030 | 0.050 |
|  | GT11 | 0.270 ± 0.027 | 0.116 ± 0.032 | 0.010 |
|  | GT14 | 0.218 ± 0.049 | 0.088 ± 0.039 | 0.021 |
|  | GT2 | 11.860 ± 0.360 | 8.656 ± 0.448 | ＜0.001 |
|  | GT2 | 0.022 ± 0.015 | 0.000 ± 0.000 | 0.002 |
|  | GT21 | 0.092 ± 0.009 | 0.019 ± 0.003 | ＜0.001 |
|  | GT23 | 0.149 ± 0.016 | 0.092 ± 0.019 | 0.028 |
|  | GT26 | 0.394 ± 0.038 | 0.128 ± 0.011 | ＜0.001 |
|  | GT28 | 1.244 ± 0.108 | 0.951 ± 0.056 | 0.028 |
|  | GT29 | 0.000 ± 0.000 | 0.001 ± 0.000 | 0.025 |
|  | GT3 | 0.242 ± 0.012 | 0.350 ± 0.037 | 0.050 |
|  | GT32 | 0.572 ± 0.067 | 0.305 ± 0.058 | 0.021 |
|  | GT5 | 0.857 ± 0.030 | 0.977 ± 0.037 | 0.028 |
|  | GT53 | 0.000 ± 0.000 | 0.001 ± 0.000 | ＜0.001 |
|  | GT56 | 0.011 ± 0.008 | 0.062 ± 0.028 | 0.005 |
|  | GT64 | 0.001 ± 0.001 | 0.001 ± 0.000 | 0.031 |
|  | GT7 | 0.035 ± 0.015 | 0.005 ± 0.001 | 0.010 |
|  | GT8 | 0.815 ± 0.053 | 0.435 ± 0.082 | ＜0.001 |
|  | GT9 | 0.906 ± 0.138 | 0.544 ± 0.114 | 0.021 |
|  | GT90 | 0.054 ± 0.014 | 0.027 ± 0.010 | 0.028 |
|  | GT94 | 0.111 ± 0.012 | 0.241 ± 0.029 | 0.002 |
|  | GT97 | 0.001 ± 0.001 | 0.001 ± 0.000 | 0.015 |
| PL | PL1 | 0.742 ± 0.092 | 0.193 ± 0.034 | ＜0.001 |
|  | PL11 | 0.379 ± 0.037 | 0.154 ± 0.033 | 0.002 |
|  | PL14 | 0.001 ± 0.001 | 0.006 ± 0.002 | 0.010 |
|  | PL18 | 0.006 ± 0.006 | 0.008 ± 0.005 | 0.013 |
|  | PL20 | 0.000 ± 0.000 | 0.001 ± 0.000 | 0.018 |
|  | PL21 | 0.004 ± 0.001 | 0.034 ± 0.011 | 0.001 |
|  | PL22 | 0.352 ± 0.021 | 0.885 ± 0.138 | ＜0.001 |
|  | PL4 | 0.016 ± 0.005 | 0.001 ± 0.000 | ＜0.001 |
|  | PL7 | 0.005 ± 0.001 | 0.000 ± 0.000 | ＜0.001 |

The values shown are means ± SEM (standard error of the mean); *P* < 0.05 indicated that mean values were significantly different. CON, nutritionally enriched group; LN, solid diet deficiency group received only milk.

**
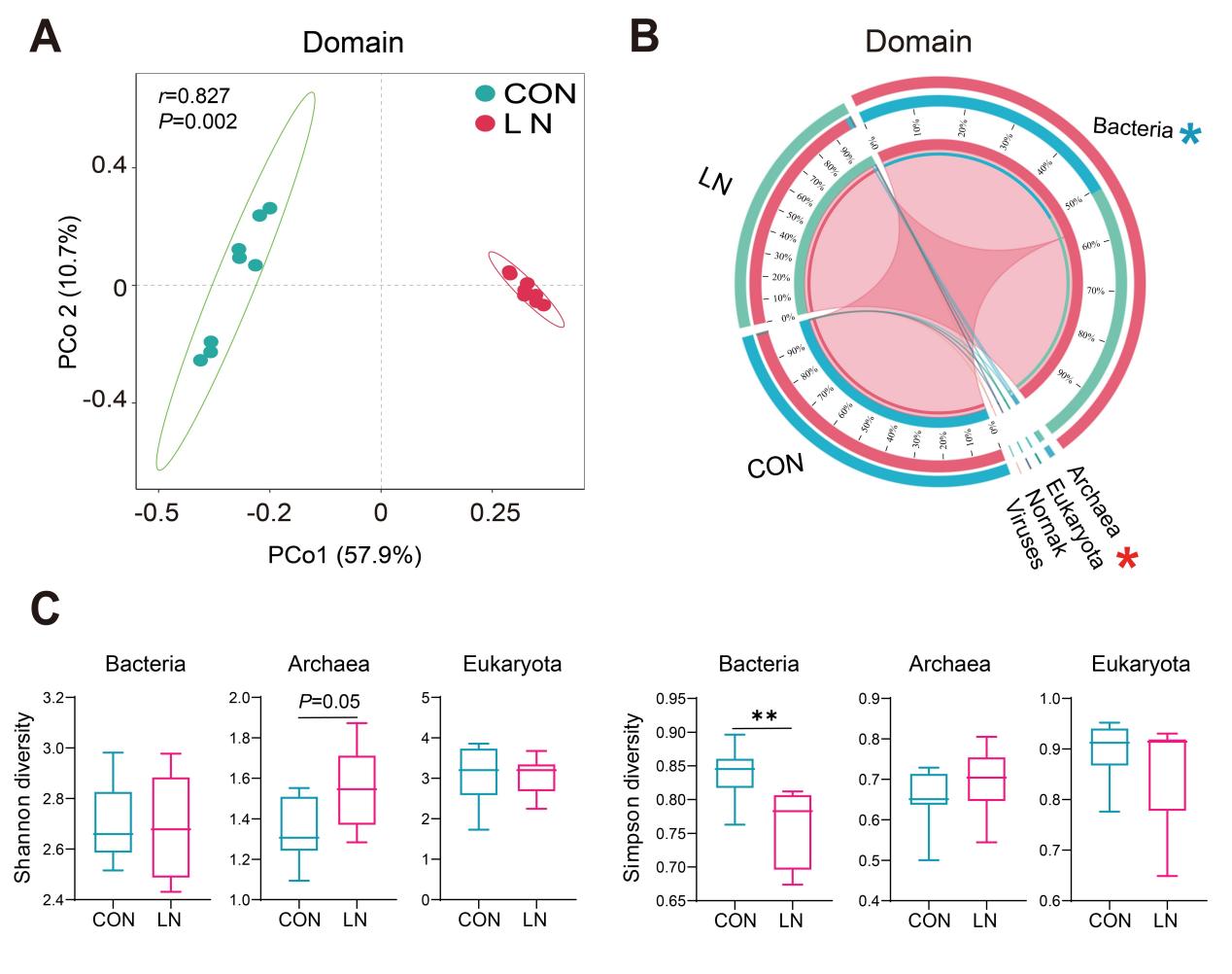
Fig. S1** Microbial structure and diversity at the domain level. A: PCoA score plot. B: Composition and differences of microbes at the domain level. C: Alpha diversity of bacteria, archaea, and eukaryotes. Blue asterisks denote higher microbial abundance in CON group, while red asterisks denote higher microbial abundance in LN group. CON, nutritionally enriched group; LN, solid diet deficiency group received only milk.

**
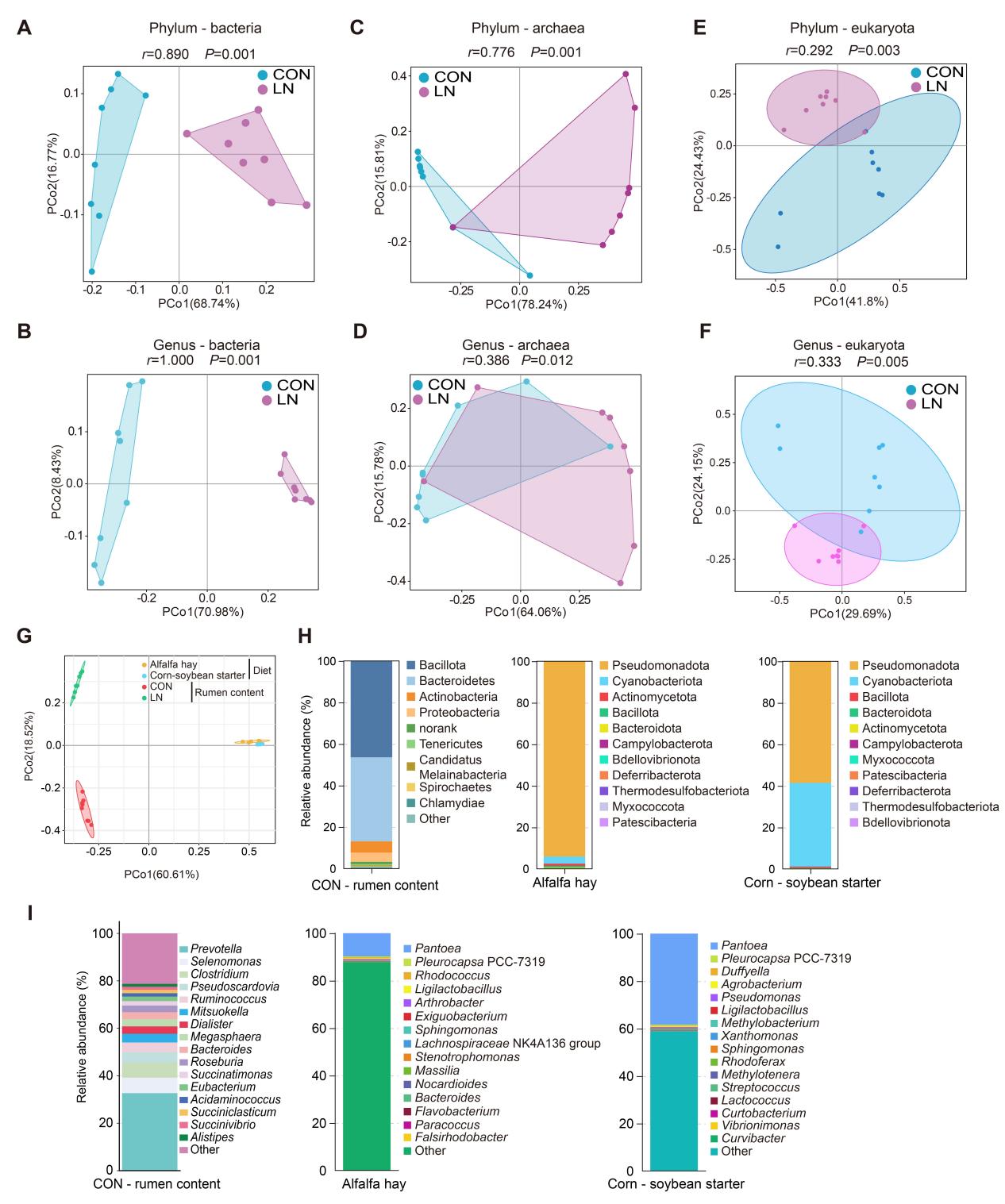
Fig. S2** Principal Coordinates Analysis (PCoA) based on unweighted UniFrac at the phylum level and genus level of microbiota and bacteria composition attached to the diet. A: Bacterial phylum level; B: Bacterial genus level; C: Archaeal phylum level; D: Archaeal genus level; E: Eukaryotic phylum level; F: Eukaryotic genus level. G: PCoA based on unweighted UniFrac at the genus level of bacteria composition of the content and diet. H: The phylum-level composition of bacteria attached to the CON-rumen content and diet. I: The genus-level composition of bacteria attached to the CON-rumen content and diet. CON, nutritionally enriched group; LN, solid diet deficiency group received only milk.

**
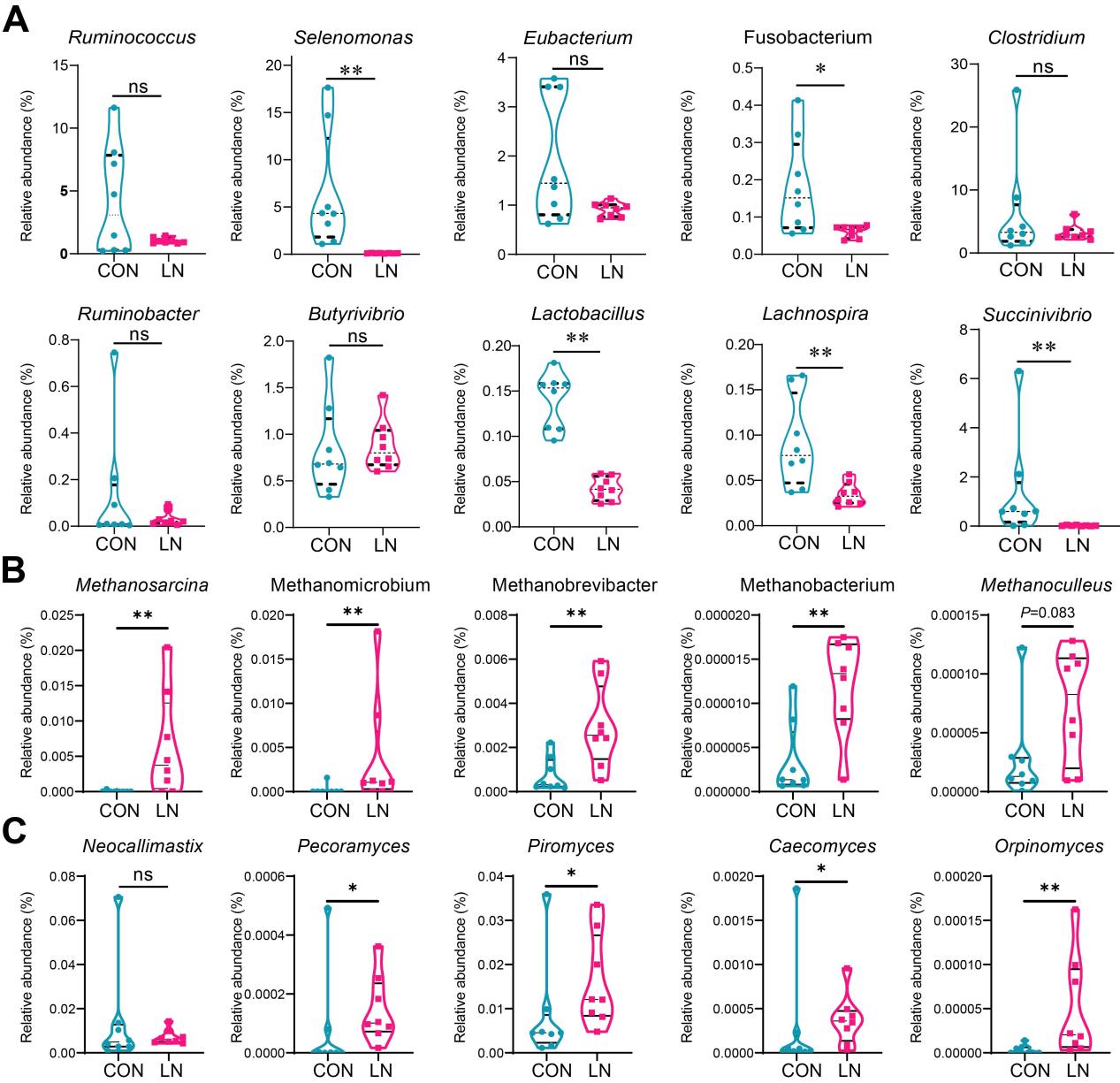
Fig. S3** Comparative analysis of microbial abundance associated with nitrogen and energy metabolism. A: Bacteria, B: Archaea, C: Eukaryotes. “*” represents *P* < 0.05, “**” represents *P* < 0.01; CON, nutritionally enriched group; LN, solid diet deficiency group received only milk. The data are presented as the mean ± SEM.

**
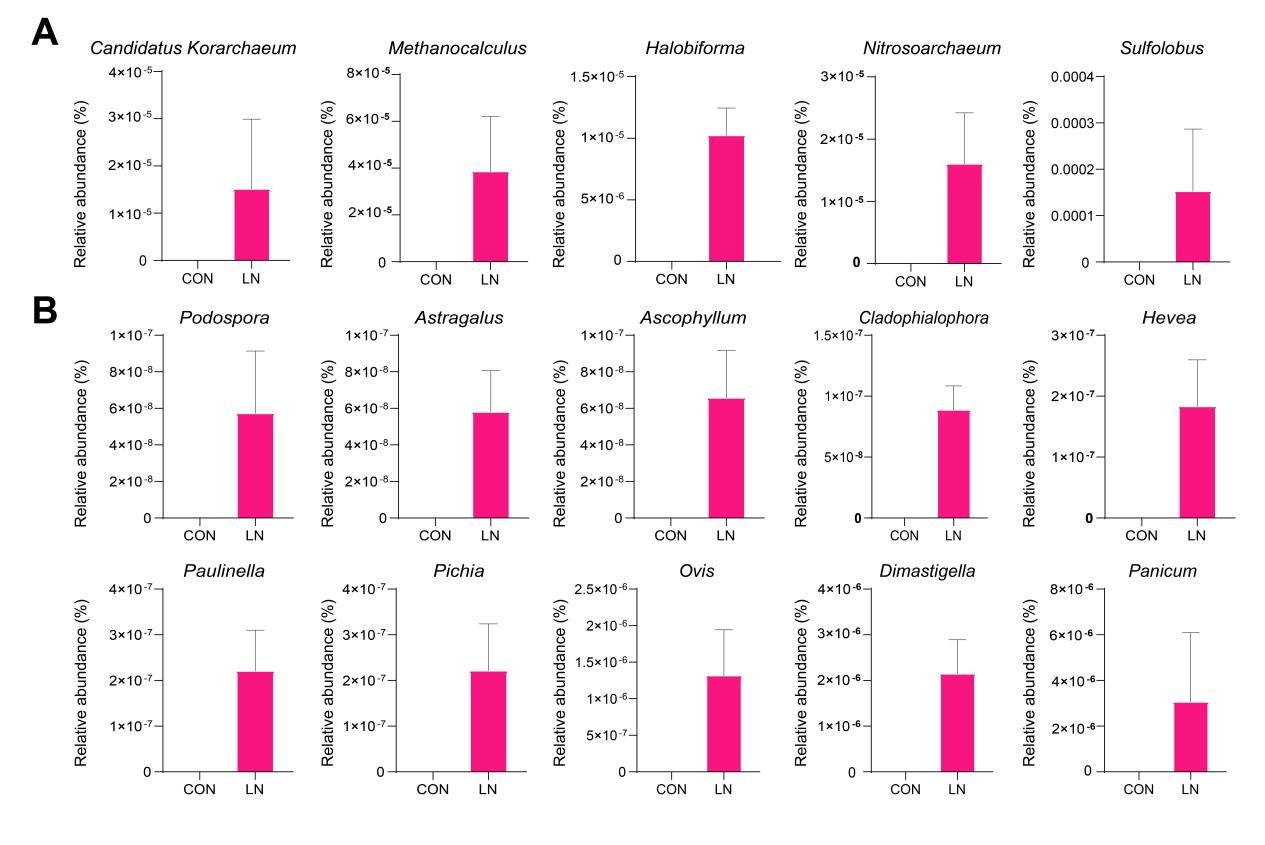
Fig. S4** Distinctive relative abundance of archaea (A) and eukaryotes (B) with solid diet deficiency. CON, nutritionally enriched group; LN, solid diet deficiency group received only milk. The data are presented as the mean±SEM.

**
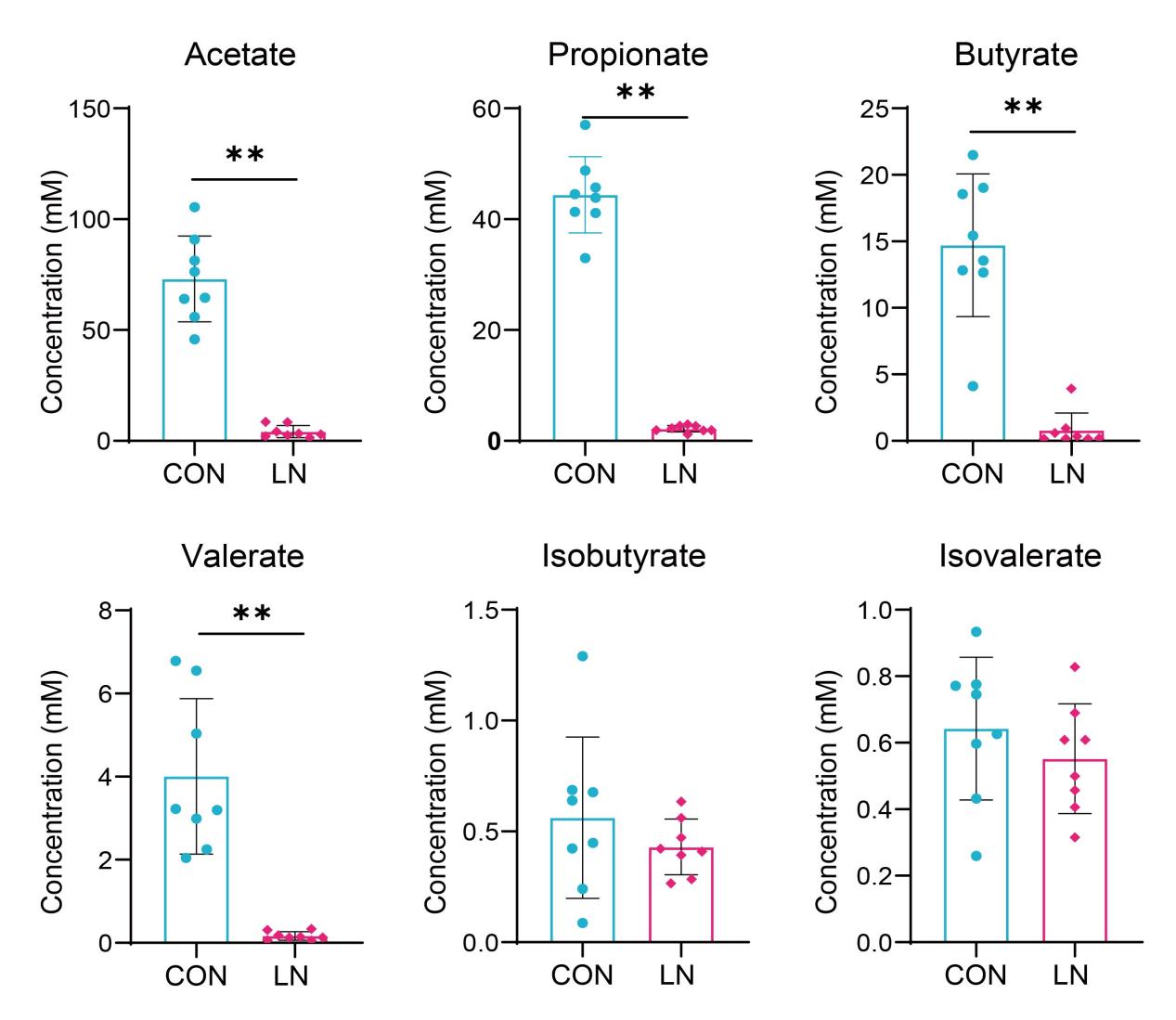
Fig. S5** Comparative analysis of concentrations of ruminal volatile fatty acid between CON and LN groups. “*” represents *P* < 0.05, “**” represents *P* < 0.01; CON, nutritionally enriched group; LN, solid diet deficiency group received only milk. The data are presented as the mean±SEM.

**
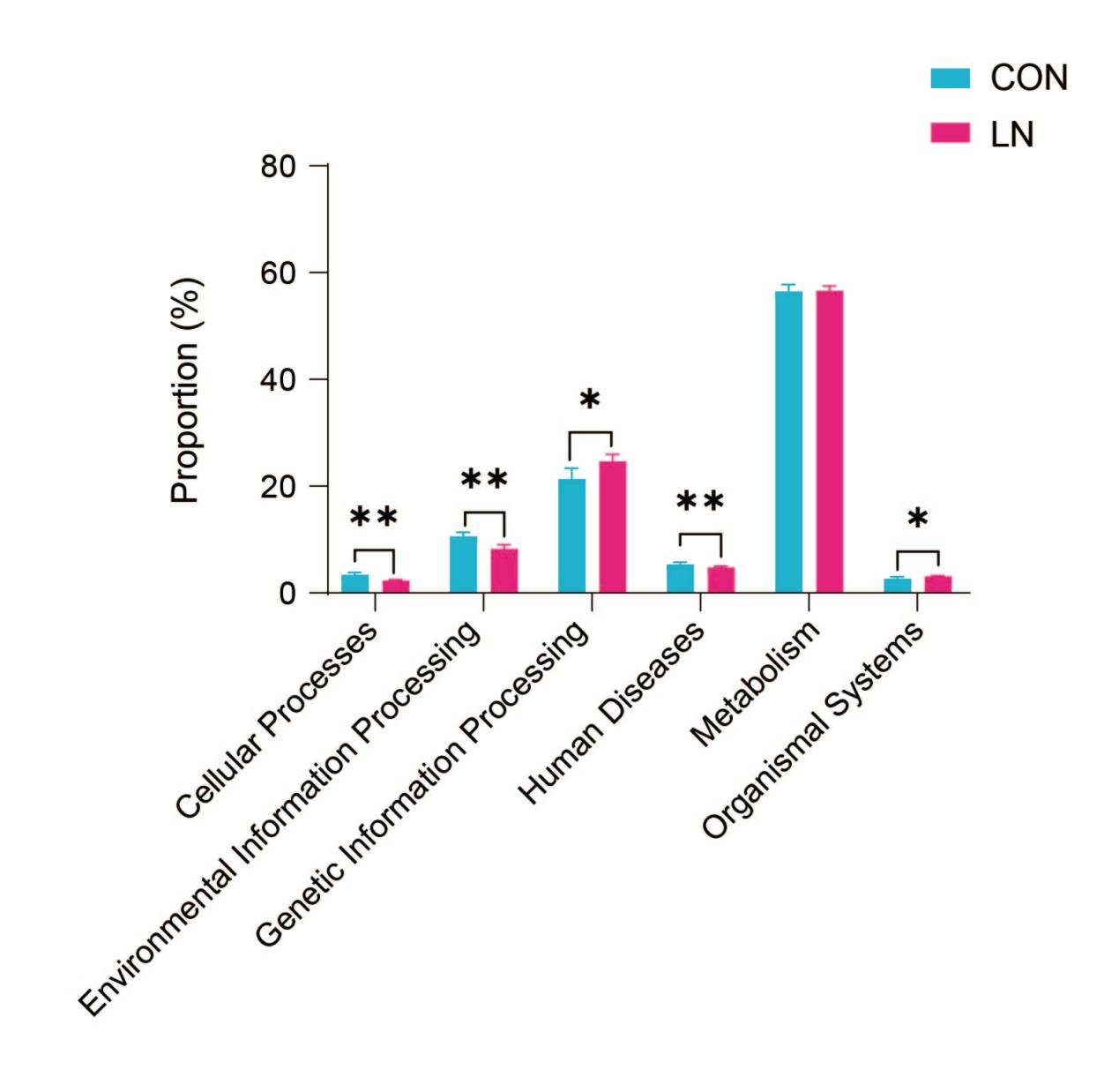
Fig. S6** Differential metabolic pathways of rumen microbiota at KEGG level 1. “*” represents *P* < 0.05, “**” represents *P* < 0.01; CON, nutritionally enriched group; LN, solid diet deficiency group received only milk. The data are presented as the mean ± SEM.

**
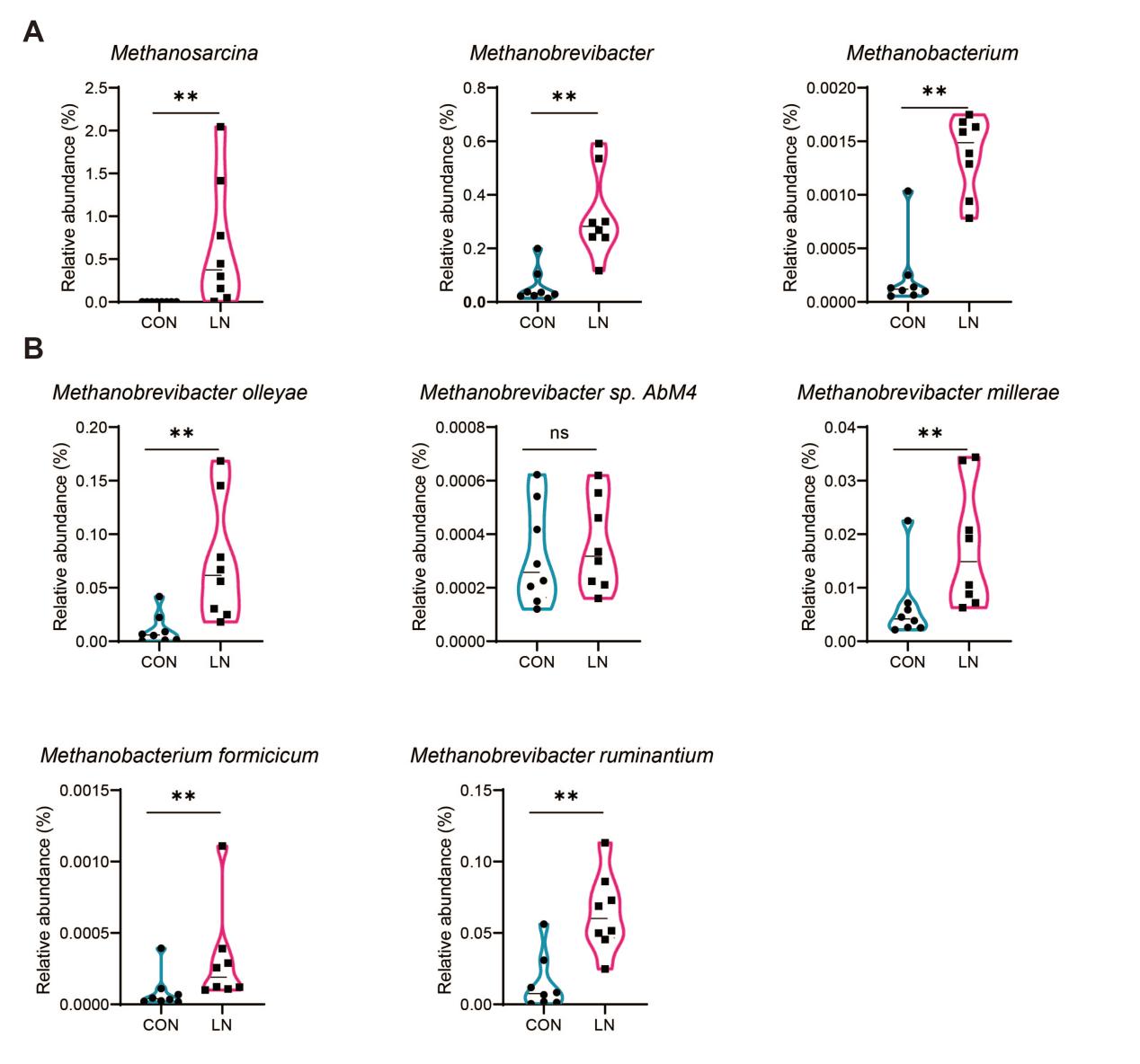
Fig. S7** The comparison of the relative abundance of archaea capable of scavenging hydrogen at genus level (A) and species level (B) “*” represents *P* < 0.05, “**” represents *P* < 0.01; CON, nutritionally enriched group; LN, solid diet deficiency group received only milk. The data are presented as the mean ± SEM.

**
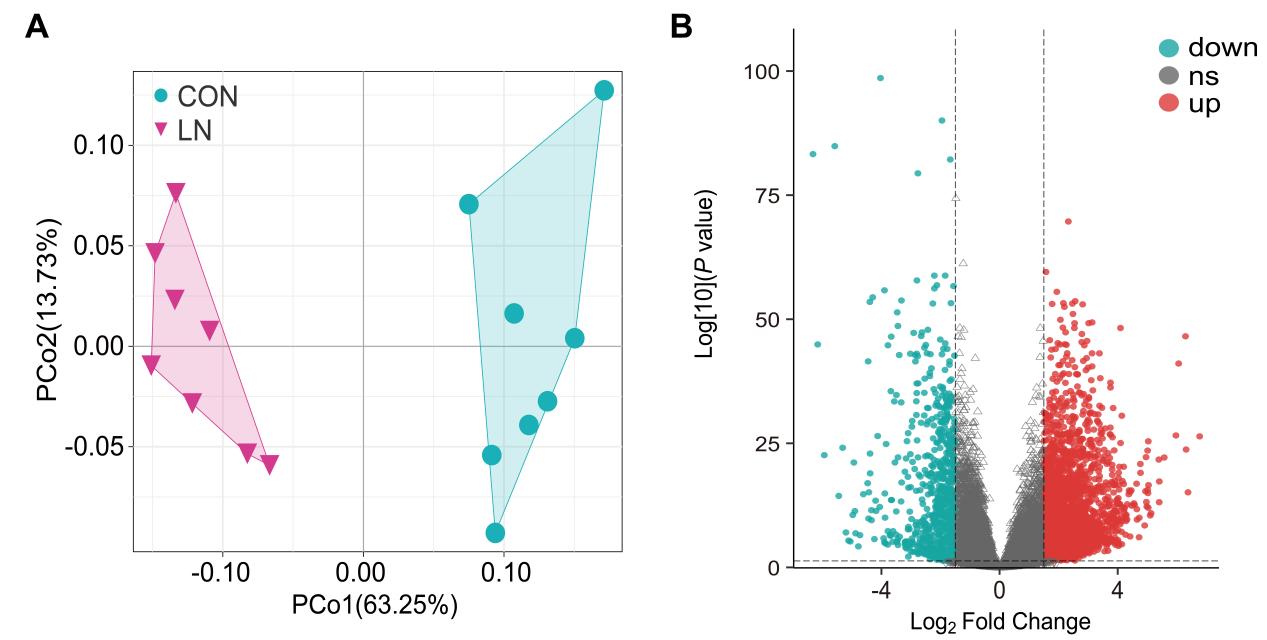
Fig. S8** Transcriptional profiles of rumen epithelia. A: Composition map of epithelial genes based on PCoA. B: Volcano plot of differential genes. CON, nutritionally enriched group; LN, solid diet deficiency group received only milk.
